# Supplementary material for: Institutional dashboards on clinical trial transparency for University Medical Centers: A case study
Source: PLoS Med. 2023 Mar 21;20(3):e1004175. doi: 10.1371/journal.pmed.1004175 (PMC10030018; doi:10.1371/journal.pmed.1004175)
Supplement: S1 Supplement — (PDF) [file pmed.1004175.s001.pdf]

# S1 Supplement: Use of automated vs manual approaches across methods

| Steps | Automated vs manual | More details | Resources/code used |
|---|---|---|---|
| Generate institutional cohort of clinical trials | Semi-automated | - We used automated methods to search registries for clinical trials associated with a given institution<br>- We performed a manual check of all trial affiliations to remove false positives | - ClinicalTrials.gov: AACT relational database<br>- DRKS: download from the registry |
| Find results publications associated with these clinical trials | Manual | - We performed a manual search for results publications associated to trials using several search engines (e.g., Google Scholar)<br>- In some cases, the results publications of a trial are linked in the registry. We used automated methods to identify such publications. However, a manual check was still needed to confirm these publications do in fact report the results of the trial | - More details on the publication search:<br>- Protocols: https://osf.io/fh426/, https://osf.io/98j7u/;<br>- Publications: IntoValue 1 study; IntoValue 2 study |
| Assess prospective registration | Automated | - Automated methods were used to obtain this information from the registry | - ClinicalTrials.gov AACT API (https://github.com/maia-sh/intovalue-data; https://github.com/maia-sh/aactr)<br>- DRKS: custom-made web scraper (https://github.com/maia-sh/intovalue-data) |
| Assess reporting of summary results in the registry | Automated | - Automated methods were used to obtain this information from the registry<br>- Summary results reporting on the European Union Clinical Trials Register: this was obtained from the EU Trials Tracker using automated methods | - ClinicalTrials.gov AACT API (https://github.com/maia-sh/intovalue-data; https://github.com/maia-sh/aactr)<br>- DRKS: custom-made web scraper (https://github.com/maia-sh/intovalue-data)<br>- EU Trials Tracker: https://eu.trialstracker.net |
| Assess linked publications | Automated | - Automated methods were used to obtain this information from the registry | - ClinicalTrials.gov AACT API (https://github.com/maia-sh/intovalue-data; https://github.com/maia-sh/aactr) |

| | | | |
|---|---|---|---|
| in the registry | | | - DRKS: custom-made web scraper (https://github.com/maia-sh/intovalue-data) |
| Assess reporting of trial registration numbers in publication abstract and main text | Automated | - Automated methods were used to obtain the publication abstracts from PubMed<br><br>- Automated methods were used to obtain the full-text PDFs from journals. Manual methods were used for few articles which could not be fetched automatically<br><br>- Automated methods were used to parse PDFs into machine-readable XMLs<br><br>- Automated methods were used to search the TRN in the publication abstract and main text | - PubMed API (https://github.com/maia-sh/intovalue-data; https://github.com/ropensci/rentrez)<br><br>- Full text (https://github.com/maia-sh/intovalue-data)<br><br>- GROBID PDF converter (https://github.com/kermitt2/grobid)<br><br>- Regular expressions for trial registration numbers from ctregistries (https://github.com/maia-sh/ctregistries) |
| Assess open access | Automated | - Query Unpaywall via its API | - UnpaywallR (https://github.com/quest-bih/unpaywallR) |
| Generate dashboard | Automated | - Shiny Dashboard (to be adapted as needed) | - Code repository (https://github.com/quest-bih/clinical-dashboard) |
