# Supplementary material for: Institutional dashboards on clinical trial transparency for University Medical Centers: A case study
Source: PLoS Med. 2023 Mar 21;20(3):e1004175. doi: 10.1371/journal.pmed.1004175 (PMC10030018; doi:10.1371/journal.pmed.1004175)
Supplement: S2 Supplement — (PDF) [file pmed.1004175.s002.pdf]

S2 Supplement: Inclusion and exclusion criteria

IntoValue dataset:

- Study type: interventional (per the registration on the retrieval date)
- Study status:
  - ClinicalTrials.gov: “Completed”; “Terminated”; “Suspended”; “Unknown” (per the registration on the retrieval date)
  - DRKS: "Recruiting complete, follow-up complete"; "Recruiting stopped after recruiting started"; "Recruiting suspended on temporary hold" (per the registration on the retrieval date)
- Study completion date between 2009 – 2017 (per the registration on the retrieval date)
- Led by one of the 35 German UMCs considered in this study (i.e., either as sponsor, responsible party, or as host of the principal investigator). Kiel and Lübeck are represented as a single UMC, namely Schleswig-Holstein.

Note on deduplication of cross-registered trials in IntoValue: we deduplicated trials in the IntoValue cohort that were cross-registered in both ClinicalTrials.gov and DRKS. If the DRKS registration mentioned a ClinicalTrials.gov identifier as a secondary ID, we favored the registration in ClinicalTrials.gov (https://github.com/quest-bih/IntoValue2/blob/master/code/1_sample_generation/Create_DRKS_sample.R#L110). We recognize that there could be additional cross-registrations that were missed in the automated check.

Updated dataset for prospective registration in ClinicalTrials.gov:

- Study type: interventional (per the registration on the retrieval date)
- Study status:
  - Status: “Completed”; “Terminated”; “Suspended”; “Unknown” (per the registration on the retrieval date)
- Study start date between 2006 – 2018 (per the registration on the retrieval date)
- Led by one of the 35 German UMCs considered in this study (i.e., either as sponsor, responsible party, or as host of the principal investigator). Kiel and Lübeck are represented as a single UMC, namely Schleswig-Holstein.
