# Supplementary material for: Institutional dashboards on clinical trial transparency for University Medical Centers: A case study
Source: PLoS Med. 2023 Mar 21;20(3):e1004175. doi: 10.1371/journal.pmed.1004175 (PMC10030018; doi:10.1371/journal.pmed.1004175)
Supplement: S3 Supplement — (PDF) [file pmed.1004175.s003.pdf]

S3 Supplement: Selected sponsor names in the EU Trials Tracker

For UMCs with more than one corresponding sponsor name in the EU Trials Tracker, we selected the sponsor name with the most trials in the EUCTR. Augsburg, Bielefeld, and Oldenburg were not included for parity with IntoValue. For Mannheim, no corresponding sponsor name was found (August 2021). Abbreviations: EUTT: EU Trials Tracker; RWTH: Rheinisch-Westfälische Technische Hochschule Aachen; UMC: University Medical Center.

UMC	Relevant name(s) in EUTT	Selected name in EUTT	Notes
**Aachen**	RWTH Aachen University	RWTH Aachen University	
**Augsburg**	Klinikum Augsburg	N/A	Not included in this study
**Berlin**	Charité-Universitätsmedizin Berlin	Charité-Universitätsmedizin Berlin	
**Bielefeld**	N/A	N/A	Not included in this study
**Bochum**	Ruhr University Bochum; Universitätsklinikum Knappschaftskrankenhaus Bochum	Ruhr University Bochum	
**Bonn**	University of Bonn	University of Bonn	
**Dresden**	Dresden University of Technology	Dresden University of Technology	
**Duisburg-Essen**	University Duisburg-Essen	University Duisburg-Essen	
**Düsseldorf**	Heinrich Heine University Düsseldorf	Heinrich Heine University Düsseldorf	
**Erlangen**	University Erlangen-Nuremberg	University Erlangen-Nuremberg	
**Frankfurt**	Goethe University	Goethe University	
**Freiburg**	University of Freiburg	University of Freiburg	
**Giessen**	University of Giessen; University Hospital Giessen and Marburg	University of Giessen	
**Göttingen**	University of Göttingen	University of Göttingen	
**Greifswald**	Medical University Greifswald	Medical University Greifswald	
**Halle-Wittenberg**	Martin Luther University Halle-Wittenberg	Martin Luther University Halle-Wittenberg	
**Hamburg**	University of Hamburg	University of Hamburg	
**Hannover**	Hannover Medical School	Hannover Medical School	
**Heidelberg**	Heidelberg University Hospital; Heidelberg University; Renal Clinic Heidelberg	Heidelberg University Hospital	

**Homburg**	Saarland University	Saarland University	
**Jena**	Friedrich Schiller University Jena	Friedrich Schiller University Jena	
**Kiel**	Schleswig-Holstein University Hospital; University of Kiel	Schleswig-Holstein University Hospital	Kiel and Lübeck are represented as a single UMC (Schleswig-Holstein)
**Köln**	University of Cologne	University of Cologne	
**Leipzig**	Leipzig University	Leipzig University	
**Lübeck**	Schleswig-Holstein University Hospital	Schleswig-Holstein University Hospital	Kiel and Lübeck are represented as a single UMC (Schleswig-Holstein)
**Magdeburg**	Otto von Guericke University Magdeburg	Otto von Guericke University Magdeburg	
**Mainz**	Johannes Gutenberg University of Mainz	Johannes Gutenberg University of Mainz	
**Mannheim**	N/A	N/A	No sponsor name found
**Marburg**	Philipps-University Marburg; University Hospital Giessen and Marburg	Philipps-University Marburg	
**München LMU**	University of Munich (Ludwig-Maximilians)	University of Munich (Ludwig-Maximilians)	
**München TU**	Technical University of Munich	Technical University of Munich	
**Münster**	University of Münster	University of Münster	
**Oldenburg**	N/A	N/A	Not included in this study
**Regensburg**	University of Regensburg	University of Regensburg	
**Rostock**	Universität Rostock	Universität Rostock	
**Tübingen**	University Hospital Tübingen; Eberhard Karls University Tübingen	University Hospital Tübingen	
**Ulm**	University of Ulm	University of Ulm	
**Witten/Herdecke**	University of Witten/Herdecke	University of Witten/Herdecke	
**Würzburg**	Julius Maximilian University of Würzburg	Julius Maximilian University of Würzburg	
