# Supplementary material for: Institutional dashboards on clinical trial transparency for University Medical Centers: A case study
Source: PLoS Med. 2023 Mar 21;20(3):e1004175. doi: 10.1371/journal.pmed.1004175 (PMC10030018; doi:10.1371/journal.pmed.1004175)
Supplement: S4 Supplement — (PDF) [file pmed.1004175.s004.pdf]

S4 Supplement: Sources for Fig 1

The table below provides links to the guidelines and laws included in Fig 1. Note that this is not meant as a comprehensive list of applicable guidelines and/or laws and is tailored to the context in Germany. Further guidelines and/or laws may apply depending on the jurisdiction and characteristics of the trial. Links were retrieved in November 2022.

Practice	Provenance of guideline and/or legal regulation	Link
Prospective registration	World Medical Association (WMA)	https://www.wma.net/policies-post/wma-declaration-of-helsinki-ethical-principles-for-medical-research-involving-human-subjects/
	International Committee of Medical Journal Editors (ICMJE)	https://www.icmje.org/recommendations/browse/publishing-and-editorial-issues/clinical-trial-registration.html#one
	World Health Organization (WHO)	https://www.who.int/news/item/18-05-2017-joint-statement-on-registration
	CIOMS	https://cioms.ch/wp-content/uploads/2017/01/WEB-CIOMS-EthicalGuidelines.pdf
	EU-V 536/2014 (for Clinical Trial of an Investigational Medicinal Product)	https://eur-lex.europa.eu/legal-content/DE/TXT/PDF/?uri=CELEX:32014R0536
Summary results reporting in the registry	World Health Organization (WHO)	https://www.who.int/news/item/18-05-2017-joint-statement-on-registration
	Bundesministerium für Bildung und Forschung (BMBF)	https://projekttraeger.dlr.de/media/gesundheit/GF/Grundsaetze_Verantwortlichkeiten_Klinische_Studien.pdf
	Deutsche Forschungsgemeinschaft (DFG)	https://www.dfg.de/download/pdf/dfg_im_profil/geschaeftsstelle/publikationen/stellungnahmen_papiere/2018/181025_stellungnahme_ag_klinische_studien.pdf
	2012/C 302/03 (EU) (for Clinical Trial of an Investigational Medicinal Product)	https://eur-lex.europa.eu/legal-content/EN/TXT/PDF/?uri=CELEX:52012XC1006(01)&from=EN
Publication linkage in the registry	World Health Organization (WHO)	https://www.who.int/publications/i/item/international-standards-for-clinical-trial-registers
Results reporting as a publication	World Health Organization (WHO)	https://www.who.int/news/item/18-05-2017-joint-statement-on-registration
	Bundesministerium für Bildung und Forschung (BMBF)	https://projekttraeger.dlr.de/media/gesundheit/GF/Grundsaetze_Verantwortlichkeiten_Klinische_Studien.pdf

	Deutsche Forschungsgemeinschaft (DFG)	https://www.dfg.de/download/pdf/dfg_im_profil/geschaeftsstelle/publikationen/stellungnahmen_papiere/2018/181025_stellungnahme_ag_klinische_studien.pdf
Open Access	World Health Organization (WHO)	https://www.who.int/news/item/18-05-2017-joint-statement-on-registration
Registry linkage in the publication	Consolidated Standards of Reporting Trials (CONSORT)	http://www.consort-statement.org/downloads
	International Committee of Medical Journal Editors (ICMJE)	https://www.icmje.org/icmje-recommendations.pdf
	World Health Organization (WHO)	https://www.who.int/news/item/18-05-2017-joint-statement-on-registration
