# Supplementary material for: Institutional dashboards on clinical trial transparency for University Medical Centers: A case study
Source: PLoS Med. 2023 Mar 21;20(3):e1004175. doi: 10.1371/journal.pmed.1004175 (PMC10030018; doi:10.1371/journal.pmed.1004175)
Supplement: S5 Supplement — (PDF) [file pmed.1004175.s005.pdf]

S5 Supplement: Detailed methods and limitations of registration and reporting metrics

Prospective registration

Methods

This analysis was limited to trials registered in ClinicalTrials.gov or DRKS with a start date given in the registry. We used 2 data sources:

1. For prospective registration in DRKS, we included trials in the IntoValue cohort registered in DRKS (interventional, led by a German UMC, study completion date between 2009 and 2017, and considered as complete based on the study status in the registry – see also **S2 Supplement**);
2. For prospective registration in ClinicalTrials.gov, we used a recent cohort of interventional trials registered in ClinicalTrials.gov (interventional, led by a German UMC, study start date between 2006 and 2018, and considered as complete per the study status in the registry – see also **S2 Supplement**).

To assess whether a study was prospectively registered, we compared the date the study was first submitted to the registry with the start date given in the registry. We defined a trial to be prospectively registered if the trial was registered in the same or a previous month to the trial start date, as some registrations provide only a start month rather than an exact date.

Limitations

This data depends on registry entries being accurate and complete. This analysis only applies to trials with a start date in the registry.

Reporting of trial registration numbers (TRNs) in the publication abstract and full text

Methods

We developed open source R scripts to detect TRNs. Our regular-expression-based approach searches text strings for matches to TRN patterns for all PubMed-indexed and ICTRP-network registries. More information on this package and its application can be found in this publication. This analysis was limited to trials in the IntoValue dataset (registered in ClinicalTrials.gov or DRKS) for which a manuscript publication was found. The analysis was further restricted to publications indexed in PubMed (detection of TRN in abstract) or publications for which we could obtain the full text (detection of TRN in full text).

Limitations

This analysis was limited to trials with a manuscript publication indexed in PubMed (detection of TRN in abstract) or for which we could obtain the full text (detection of TRN in full text).

Publication links in the registry

Methods

This analysis was limited to trials in the IntoValue dataset (registered in ClinicalTrials.gov or DRKS) for which a manuscript publication was found. The analysis was further restricted to publications with a DOI or that are indexed in PubMed. We considered a publication "linked" if the PubMed Identifier (PMID) or DOI was included in the trial registration. We extracted the relevant fields from ClinicalTrials.gov and DRKS using automated methods (ClinicalTrials.gov: via its API; DRKS: custom-built web scraper; 1 November 2022) and used regular expressions to extract publication identifiers (DOIs and PMIDs) from these fields. More information on this approach can be found in this publication.

Limitations
This analysis was limited to trials with a manuscript publication which have a DOI or PMID (i.e., are indexed in PubMed). Publications included in the registration without a PMID or DOI (i.e., publication title and/or URL only) may have been missed.

Registry limitations
ClinicalTrials.gov includes an often-used PMID field for references. In addition, ClinicalTrials.gov automatically indexes publications from PubMed using TRN in the secondary identifier field. In contrast, DRKS includes references as a free-text field, leaving it up to trialists to enter publication identifiers.

Summary results reporting (EUCTR)

Methods
This analysis was limited to trials listed in the EU Trials Tracker (and therefore registered in the EUCTR) with a sponsor name corresponding to one of the included UMCs and due to report summary results. For each UMC in our dataset, we searched the corresponding sponsor name in the EU Trials Tracker (August 2021). With the exception of one UMC (Mannheim), we found at least one sponsor name for each UMC in the EU Trials Tracker. If more than one corresponding sponsor name was found for a given UMC, we selected the sponsor with the most trials on the EUCTR. Selected sponsor names can be found in **S3 Supplement**. We retrieved the relevant data (percent reported, total number of due trials, and total number of trials that reported results) from historical versions of the EU Trials Tracker's code repository (latest data extracted on 4 November 2022). While the EU Trials Tracker is usually updated monthly, in some cases there was more than one update within the same month. In these cases, only the latest data point within that month was displayed. Note that some trials registered in the EUCTR and captured in this analysis may be cross-registered in ClinicalTrials.gov and/or DRKS.

Limitations
We did not find a corresponding sponsor name in the EU Trials Tracker for all included UMCs (Mannheim). Several UMCs had more than one corresponding sponsor name in the EU Trials Tracker (Bochum, Giessen, Heidelberg, Kiel, Marburg, and Tübingen). Since we only selected the sponsor with the most trials on the EUCTR, some trials may have been missed for these UMCs.

Summary results reporting (ClinicalTrials.gov and DRKS)

Methods
This analysis was limited to trials in the IntoValue dataset (registered in ClinicalTrials.gov or DRKS). Summary results posting was extracted from ClinicalTrials.gov and DRKS via automated methods. ClinicalTrials.gov includes a structured summary results field. In contrast, DRKS includes summary results with other references. In the absence of a structured summary results field in DRKS, we detected summary results in this registry based on the presence of keywords (e.g., “Ergebnisbericht” or “Abschlussbericht”) in the reference title. The summary results date in DRKS was extracted manually from the registry's change history (which indicates when the summary result was uploaded).

Limitations
In contrast to ClinicalTrials.gov, DRKS does not include a structured summary results field but includes summary results with other references. In the absence of a structured summary results field in DRKS, we detected summary results in DRKS based on the presence of keywords (e.g., “Ergebnisbericht” or “Abschlussbericht”) in the reference title. We did not perform a manual review of these results.

2- and 5-year results reporting (summary results or manuscript publication)

Methods
We considered 2 years as timely reporting for both reporting routes This analysis was limited to trials in the IntoValue dataset (registered in ClinicalTrials.gov or DRKS).

- *Results reporting as summary results in the registry:* See above. When calculating the 2-year and 5-year reporting rates, we only included trials for which we had 2- and 5- years follow-up time from trial completion to the registry download date, respectively.
- *Results reporting as a manuscript publication:* This data is the result of automated and manual searches and was previously published. More information can be found in the IntoValue 1 and IntoValue 2 publications. A manual search for published results was performed, searching the registry, PubMed, and Google. If multiple results publications were found, the earliest was included. The data presented in this dashboard therefore does not reflect all result publications of a given trial. Publication dates were manually entered during publication searches. When calculating the 2-year and 5-year reporting rates, we only included trials for which we had 2- and 5- years follow-up time from trial completion to the manual publication search date, respectively.
- *Results reporting as summary results or manuscript publication:* When calculating the 2-year and 5-year reporting rates, we only included trials for which we had 2- and 5-years follow-up time (1) from trial completion to the registry download date **AND** (2) from trial completion to the manual search date.

Limitations

- *Results reporting as summary results in the registry:* See above. Registry data was downloaded on 1 November 2022. Summary results posted in the registry after this date would have been missed.
- *Results reporting as a manuscript publication:* The manual searches for trial results publications in the IntoValue 1 cohort (trials completed between 2009 – 2013) were performed between July 2017 and December 2017. The manual searches for trial results publications in the IntoValue 2 cohort (trials completed between 2014 – 2017) were performed between July 2020 and September 2020. Trial results publications published after these manual searches were conducted would have been missed. Furthermore, some publications may have been missed in the manual search procedure as the search was restricted to a limited number of scientific databases and the responsible parties were not contacted.

Open Access (OA) status

Methods

This analysis was limited to trials in the IntoValue dataset (registered in ClinicalTrials.gov or DRKS) with a manuscript publication. Since a DOI is needed to query Unpaywall, this analysis was further limited to publications with a DOI. The publication date from Unpaywall was used to display the data over time. Therefore, this analysis was also limited to publications with a publication date in Unpaywall. Using the publication DOIs, we queried the Unpaywall database via its API using the UnpaywallR R package to obtain information on the OA status of publications. Unpaywall harvests content from legal sources, such as publishers, repositories, and preprint servers, and has limited coverage of personal websites. It does not harvest content from academic social networks for which concerns have been raised about the persistence of content. Thus, the following definition of OA was used in this study: articles that are free to read online in a journal or OA repository. Publications can have different OA statuses which are color-coded. Gold OA denotes a publication in an OA journal. Green OA denotes a freely available repository version. Hybrid OA denotes an OA publication in a journal which offers both a subscription-based model as well as an OA option. Bronze OA denotes publications which are freely available on the journal page, but without a clear open license. These can be articles in a non-OA journal which have been made available voluntarily by the journal, but which might at some stage lose their OA status again. As publications can have several OA versions (e.g., a gold version in an OA journal as well as a green version in a repository), we defined a hierarchy for categories and for each publication only assigned the category with the highest priority. We used a hierarchy of gold - hybrid – green – bronze – closed. OA status is not fixed but rather changes over time, as repository versions are often made available with a delay. Therefore, the OA percentage for a given year typically rises

retrospectively. Thus, the point in time at which the OA status is retrieved is important for the OA percentage. Unpaywall API query date: 1 November 2022.

Limitations

The OA status could only be obtained for publications with a DOI and which resolved in Unpaywall. We did not perform a manual check of the OA data.

Unpaywall has previously been shown to provide a conservative estimate of the actual percentage of OA in the literature (see: https://peerj.com/articles/4375/). Therefore, some publications reported as closed may in fact have been openly accessible. Finally, based on the OA definition used in this study, publications that were only free to read in a location other than a journal or repository (e.g., ResearchGate) were not considered as OA.
