# Supplementary material for: Institutional dashboards on clinical trial transparency for University Medical Centers: A case study
Source: PLoS Med. 2023 Mar 21;20(3):e1004175. doi: 10.1371/journal.pmed.1004175 (PMC10030018; doi:10.1371/journal.pmed.1004175)
Supplement: S6 Supplement — (PDF) [file pmed.1004175.s006.pdf]

S6 Supplement: Strengthening the Reporting of Observational Studies in Epidemiology (STROBE) checklist for cross-sectional studies

STROBE Statement—Checklist of items that should be included in reports of ***cross-sectional studies***

	Item No	Recommendation	Section (Paragraph) No
**Title and abstract**	1	(*a*) Indicate the study's design with a commonly used term in the title or the abstract	Title, Abstract
		(*b*) Provide in the abstract an informative and balanced summary of what was done and what was found	Abstract
**Introduction**			
Background/rationale	2	Explain the scientific background and rationale for the investigation being reported	Introduction
Objectives	3	State specific objectives, including any prespecified hypotheses	Introduction (5th paragraph)
**Methods**			
Study design	4	Present key elements of study design early in the paper	Methods (1st paragraph)
Setting	5	Describe the setting, locations, and relevant dates, including periods of recruitment, exposure, follow-up, and data collection	Methods (Data sources and inclusion and exclusion criteria)
Participants	6	(*a*) Give the eligibility criteria, and the sources and methods of selection of participants	Methods (Data sources and inclusion and exclusion criteria)
Variables	7	Clearly define all outcomes, exposures, predictors, potential confounders, and effect modifiers. Give diagnostic criteria, if applicable	Methods (Analysis of registration and reporting practices)
Data sources/ measurement	8*	For each variable of interest, give sources of data and details of methods of assessment (measurement). Describe comparability of assessment methods if there is more than one group	Methods (Data sources and inclusion and exclusion criteria; Analysis of registration and reporting practices)

Bias	9	Describe any efforts to address potential sources of bias	Methods (Data sources and inclusion and exclusion criteria)
Study size	10	Explain how the study size was arrived at	Methods (Data sources and inclusion and exclusion criteria)
Quantitative variables	11	Explain how quantitative variables were handled in the analyses. If applicable, describe which groupings were chosen and why	Methods (Analysis of registration and reporting practices); S5 Supplement
Statistical methods	12	(*a*) Describe all statistical methods, including those used to control for confounding	Methods (Analysis)
		(*b*) Describe any methods used to examine subgroups and interactions	Methods (Analysis)
		(*c*) Explain how missing data were addressed	S5 Supplement
		(*d*) If applicable, describe analytical methods taking account of sampling strategy	NA
		(*e*) Describe any sensitivity analyses	NA
**Results**			
Participants	13*	(a) Report numbers of individuals at each stage of study—eg numbers potentially eligible, examined for eligibility, confirmed eligible, included in the study, completing follow-up, and analysed	Results (Characteristics of trials); S8 Supplement
		(b) Give reasons for non-participation at each stage	Results (Characteristics of trials); S8 Supplement
		(c) Consider use of a flow diagram	Results (Characteristics of trials); Fig 2; S8 Supplement
Descriptive data	14*	(a) Give characteristics of study participants (eg demographic, clinical, social) and information on exposures and potential confounders	Results (Characteristics of trials), S7 Supplement
		(b) Indicate number of participants with missing data for each variable of interest	S8 Supplement

Outcome data	15*	Report numbers of outcome events or summary measures	Results (Evaluation of trial registration and reporting practices)
Main results	16	(*a*) Give unadjusted estimates and, if applicable, confounder-adjusted estimates and their precision (eg, 95% confidence interval). Make clear which confounders were adjusted for and why they were included	Results (Evaluation of trial registration and reporting practices)
		(*b*) Report category boundaries when continuous variables were categorized	NA
		(*c*) If relevant, consider translating estimates of relative risk into absolute risk for a meaningful time period	NA
Other analyses	17	Report other analyses done—eg analyses of subgroups and interactions, and sensitivity analyses	Results (Interactive dashboard)
**Discussion**			
Key results	18	Summarise key results with reference to study objectives	Discussion (1st paragraph)
Limitations	19	Discuss limitations of the study, taking into account sources of potential bias or imprecision. Discuss both direction and magnitude of any potential bias	Discussion (Limitations)
Interpretation	20	Give a cautious overall interpretation of results considering objectives, limitations, multiplicity of analyses, results from similar studies, and other relevant evidence	Discussion (Trends in trial transparency)
Generalisability	21	Discuss the generalisability (external validity) of the study results	Discussion (Actionable areas for stakeholders; Scalability beyond German UMCs; Stakeholder and community engagement; Updates and further development of the dashboard)
**Other information**			
Funding	22	Give the source of funding and the role of the funders for the present study and, if applicable,	NA

	for the original study on which the present article is based	

*Give information separately for exposed and unexposed groups.

Note: An Explanation and Elaboration article discusses each checklist item and gives methodological background and published examples of transparent reporting. The STROBE checklist is best used in conjunction with this article (freely available on the Web sites of PLoS Medicine at http://www.plosmedicine.org/, Annals of Internal Medicine at http://www.annals.org/, and Epidemiology at http://www.epidem.com/). Information on the STROBE Initiative is available at www.strobe-statement.org.
