# Supplementary material for: Institutional dashboards on clinical trial transparency for University Medical Centers: A case study
Source: PLoS Med. 2023 Mar 21;20(3):e1004175. doi: 10.1371/journal.pmed.1004175 (PMC10030018; doi:10.1371/journal.pmed.1004175)
Supplement: S7 Supplement — (PDF) [file pmed.1004175.s007.pdf]

S7 Supplement: Characteristics of included trials

Characteristics of German UMC-led trials (*IntoValue dataset)*

A trial was considered randomized if allocation included randomization. Trials were considered to be led by a German UMC if the UMC was listed in ‘sponsors’, ‘overall officials’, or ‘responsible parties’ in ClinicalTrials.gov, or in any ‘addresses’ in DRKS. 'Unknowns' are not counted in the denominator for percentages. Abbreviations: CT.gov: ClinicalTrials.gov; DRKS: German Clinical Trials Register; EUCTR: EU Clinical Trials Register; ID: identifier; IQR: interquartile range.

	**Overall, N = 2,895**	**ClinicalTrials.gov, N = 2,253**	**DRKS, N = 642**
**Randomized, n (%)**	2,001 (83%)	1,557 (88%)	444 (69%)
**Unknown**	480	479	1
**Multicentric trial, n (%)**	998 (34%)	843 (37%)	155 (24%)
**Unknown**	2	0	2
**Industry sponsor, n (%)**	47 (1.6%)	0 (0%)	47 (7.3%)
**Trial enrollment, Median (IQR)**	60 (30, 140)	60 (30, 140)	60 (30, 130)
**Unknown**	9	9	0
**EUCTR Trial ID in Registration, n (%)**	431 (15%)	345 (15%)	86 (13%)
**Phase, n (%)**			
**I**	153 (12%)	124 (11%)	29 (22%)
**I-II**	98 (7.5%)	92 (7.8%)	6 (4.7%)
**II**	444 (34%)	410 (35%)	34 (26%)
**II-III**	60 (4.6%)	57 (4.8%)	3 (2.3%)
**III**	279 (21%)	254 (22%)	25 (19%)
**IV**	272 (21%)	240 (20%)	32 (25%)
**Unknown**	1,589	1,076	513
**Intervention Type, n (%)**			
**Behavioral**	245 (8.5%)	245 (11%)	0 (0%)
**Biological**	74 (2.6%)	74 (3.3%)	0 (0%)
**Combination Product**	1 (<0.1%)	1 (<0.1%)	0 (0%)
**Device**	497 (17%)	497 (22%)	0 (0%)
**Diagnostic Test**	1 (<0.1%)	1 (<0.1%)	0 (0%)
**Dietary Supplement**	102 (3.5%)	102 (4.5%)	0 (0%)
**Drug**	815 (28%)	815 (36%)	0 (0%)
**Genetic**	3 (0.1%)	3 (0.1%)	0 (0%)
**Not given**	642 (22%)	0 (0%)	642 (100%)
**Other**	232 (8.0%)	232 (10%)	0 (0%)
**Procedure**	251 (8.7%)	251 (11%)	0 (0%)
**Radiation**	32 (1.1%)	32 (1.4%)	0 (0%)
**Trial start year, n (%)**			
**1992**	1 (<0.1%)	1 (<0.1%)	0 (0%)
**1998**	1 (<0.1%)	0 (0%)	1 (0.2%)
**1999**	1 (<0.1%)	1 (<0.1%)	0 (0%)
**2000**	3 (0.1%)	3 (0.1%)	0 (0%)
**2001**	6 (0.2%)	6 (0.3%)	0 (0%)
**2002**	21 (0.7%)	19 (0.8%)	2 (0.3%)
**2003**	19 (0.7%)	18 (0.8%)	1 (0.2%)
**2004**	37 (1.3%)	36 (1.6%)	1 (0.2%)
**2005**	43 (1.5%)	38 (1.7%)	5 (0.8%)
**2006**	89 (3.1%)	83 (3.7%)	6 (0.9%)
**2007**	139 (4.8%)	129 (5.7%)	10 (1.6%)
**2008**	229 (7.9%)	201 (8.9%)	28 (4.4%)
**2009**	285 (9.9%)	243 (11%)	42 (6.5%)
**2010**	338 (12%)	278 (12%)	60 (9.3%)
**2011**	349 (12%)	277 (12%)	72 (11%)
**2012**	338 (12%)	262 (12%)	76 (12%)
**2013**	285 (9.9%)	194 (8.6%)	91 (14%)
**2014**	270 (9.3%)	182 (8.1%)	88 (14%)

**2015**	238 (8.2%)	154 (6.8%)	84 (13%)
**2016**	151 (5.2%)	100 (4.4%)	51 (7.9%)
**2017**	49 (1.7%)	25 (1.1%)	24 (3.7%)
**Unknown**	3	3	0
**Trial completion year, n (%)**			
**2009**	151 (5.2%)	137 (6.1%)	14 (2.2%)
**2010**	227 (7.8%)	186 (8.3%)	41 (6.4%)
**2011**	278 (9.6%)	226 (10%)	52 (8.1%)
**2012**	304 (11%)	251 (11%)	53 (8.3%)
**2013**	294 (10%)	240 (11%)	54 (8.4%)
**2014**	381 (13%)	285 (13%)	96 (15%)
**2015**	438 (15%)	326 (14%)	112 (17%)
**2016**	406 (14%)	284 (13%)	122 (19%)
**2017**	416 (14%)	318 (14%)	98 (15%)

Characteristics of German UMC-led trials (***recent cohort for prospective registration in ClinicalTrials.gov)***

A trial was considered randomized if allocation included randomization. Trials were considered to be led by a German UMC if the UMC was listed in ‘sponsors’, ‘overall officials’, or ‘responsible parties’ in ClinicalTrials.gov. 'Unknowns' are not counted in the denominator for percentages.

	**ClinicalTrials.gov, N = 3,618**
**Randomized, n (%)**	2,504 (88%)
**Unknown**	770
**Multicentric trial, n (%)**	1,400 (39%)
**Industry sponsor, n (%)**	0 (0%)
**Trial enrollment, Median (IQR)**	63 (30, 155)
**Unknown**	17
**EUCTR Trial ID in Registration, n (%)**	558 (15%)
**Phase, n (%)**	
**I**	194 (11%)
**I-II**	145 (8.4%)
**II**	603 (35%)
**II-III**	78 (4.5%)
**III**	381 (22%)
**IV**	331 (19%)
**Unknown**	1,886
**Intervention Type, n (%)**	
**Behavioral**	426 (12%)
**Biological**	125 (3.5%)
**Combination Product**	3 (<0.1%)
**Device**	798 (22%)
**Diagnostic Test**	19 (0.5%)
**Dietary Supplement**	152 (4.2%)
**Drug**	1,216 (34%)
**Genetic**	5 (0.1%)
**Other**	417 (12%)
**Procedure**	390 (11%)
**Radiation**	67 (1.9%)
**Trial start year, n (%)**	
**2006**	178 (4.9%)
**2007**	230 (6.4%)
**2008**	256 (7.1%)
**2009**	273 (7.5%)
**2010**	343 (9.5%)
**2011**	359 (9.9%)
**2012**	333 (9.2%)
**2013**	289 (8.0%)
**2014**	285 (7.9%)
**2015**	310 (8.6%)
**2016**	322 (8.9%)
**2017**	247 (6.8%)
**2018**	193 (5.3%)

**Trial completion year, n (%)**	
**2006**	2 (<0.1%)
**2007**	32 (0.9%)
**2008**	91 (2.6%)
**2009**	140 (4.0%)
**2010**	182 (5.2%)
**2011**	217 (6.2%)
**2012**	258 (7.4%)
**2013**	268 (7.7%)
**2014**	286 (8.2%)
**2015**	345 (9.9%)
**2016**	301 (8.6%)
**2017**	352 (10%)
**2018**	322 (9.2%)
**2019**	269 (7.7%)
**2020**	240 (6.9%)
**2021**	132 (3.8%)
**2022**	49 (1.4%)
**2023**	1 (<0.1%)
**2024**	1 (<0.1%)
**2025**	2 (<0.1%)
**2027**	1 (<0.1%)
**Unknown**	127
