# Supplementary material for: Institutional dashboards on clinical trial transparency for University Medical Centers: A case study
Source: PLoS Med. 2023 Mar 21;20(3):e1004175. doi: 10.1371/journal.pmed.1004175 (PMC10030018; doi:10.1371/journal.pmed.1004175)

S8 Supplement: Flow diagrams of the trial and publication screening

Flowchart 1
Trial screening flowchart for the evaluation of the following practice: prospective registration. **Left:** Prospective registration in DRKS was derived from the IntoValue cohort (box with the thicker contour). The denominator for prospective registration in DRKS is highlighted in pink. **Right:** Prospective registration in ClinicalTrials.gov was derived from an updated cohort. The denominator for prospective registration in ClinicalTrials.gov is highlighted in pink. Abbreviations: CT.gov: ClinicalTrials.gov; DRKS: German Clinical Trials Register; UMC: University Medical Center.

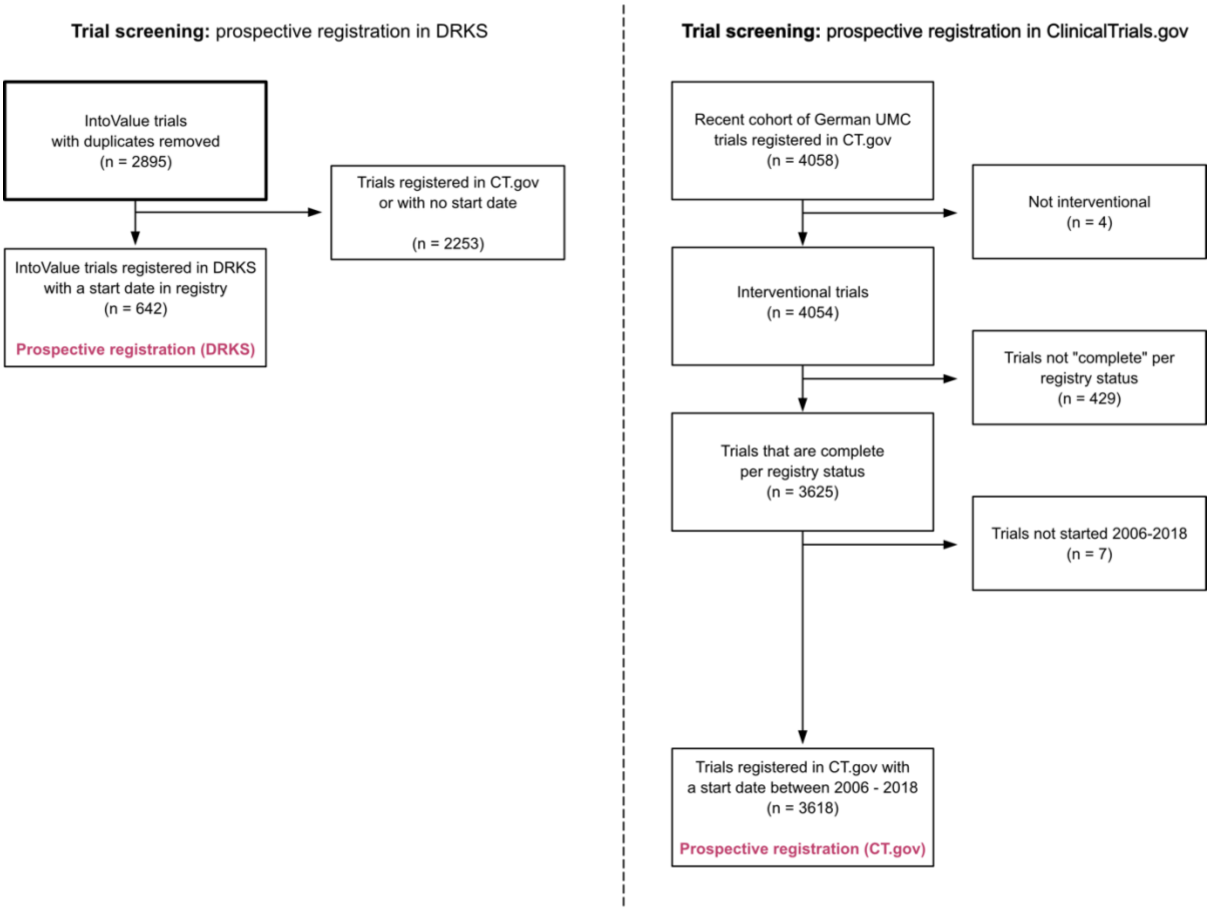

Flowchart 2

Trial screening flowchart for the evaluation of the following practices: publication link in the registry, TRN reporting in the publication abstract, and TRN reporting in the publication full text. These data were derived from the IntoValue cohort (registered in DRKS or ClinicalTrials.gov). The box with the thicker contour displays how this flowchart follows from Flowchart 1. The relevant denominators are highlighted in pink. Abbreviations: DOI: Digital Object Identifier; PMID: PubMed identifier.

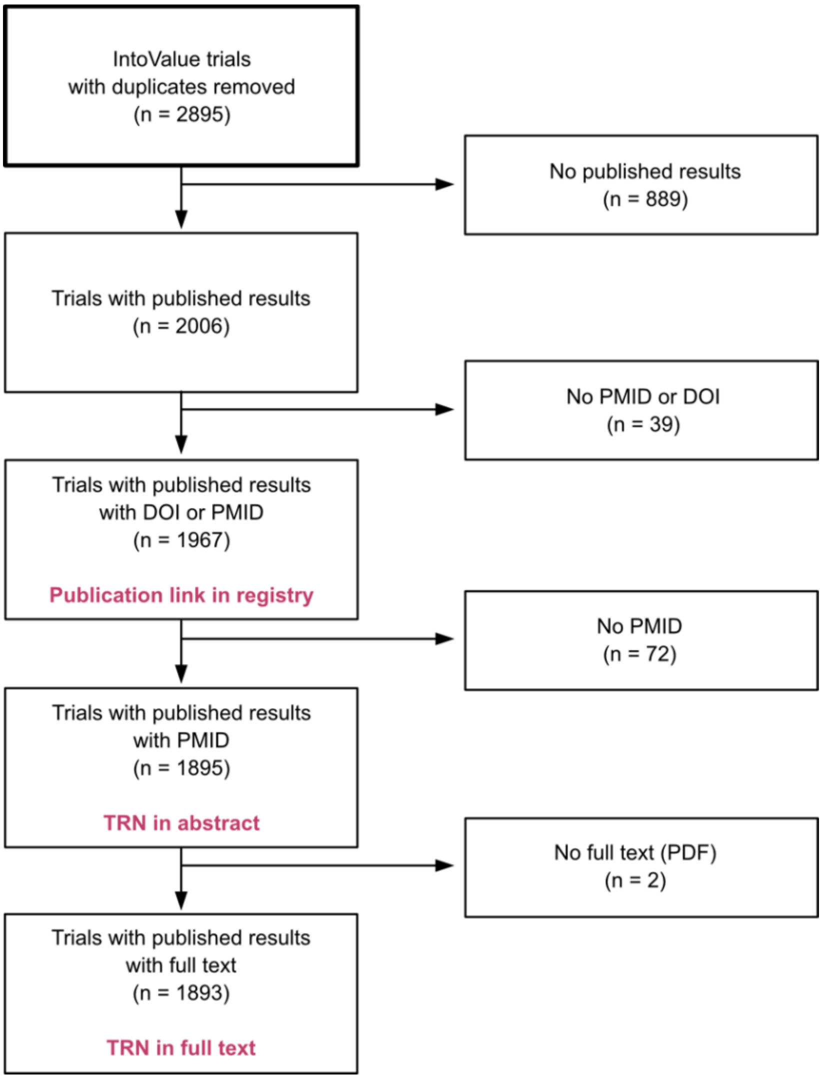

Flowchart 3

Trial screening flowchart for the evaluation of the following practices:

- Any summary results reporting for due trials
- Timeliness (within 2- and 5-years since trial completion) of the following reporting routes: a) summary results, b) manuscript publication, and c) summary results OR a manuscript publication

When calculating the 2-year and 5-year reporting rates, we only included:

- Summary results: Trials for which we had 2- and 5-years follow-up time from trial completion to the registry download date
- Manuscript publication: Trials for which we had 2- and 5-years follow-up time from trial completion to the manual search date
- Summary results OR manuscript publication: Trials for which we had 2- and 5-years follow-up time (1) from trial completion to the registry download date AND (2) from trial completion to the manual search date

These data were derived from the IntoValue cohort (registered in DRKS or ClinicalTrials.gov). The box with the thicker contour displays how this flowchart follows from Flowchart 1. The relevant denominators are highlighted in pink.

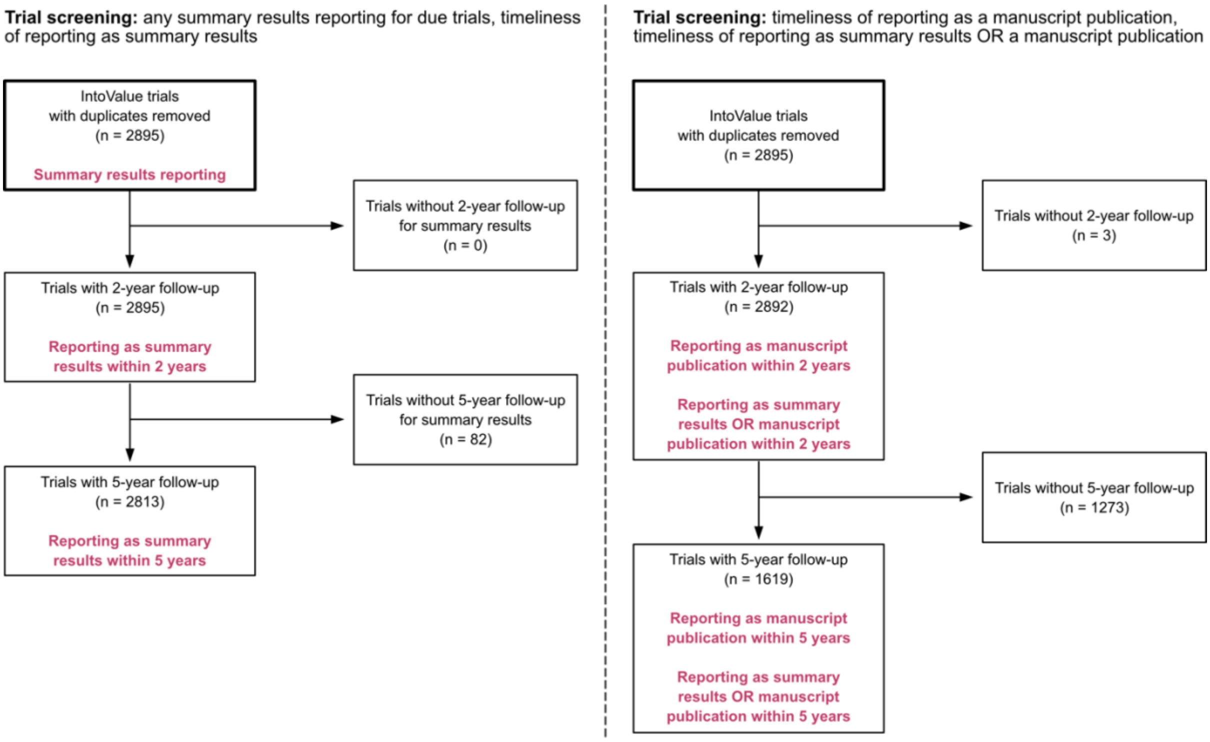

Flowchart 4

Publication screening flowchart for the evaluation of the following practice: Open Access. These data were derived from the IntoValue cohort (registered in DRKS or ClinicalTrials.gov). The relevant denominator is highlighted in pink. Abbreviations: DOI: Digital Object Identifier.

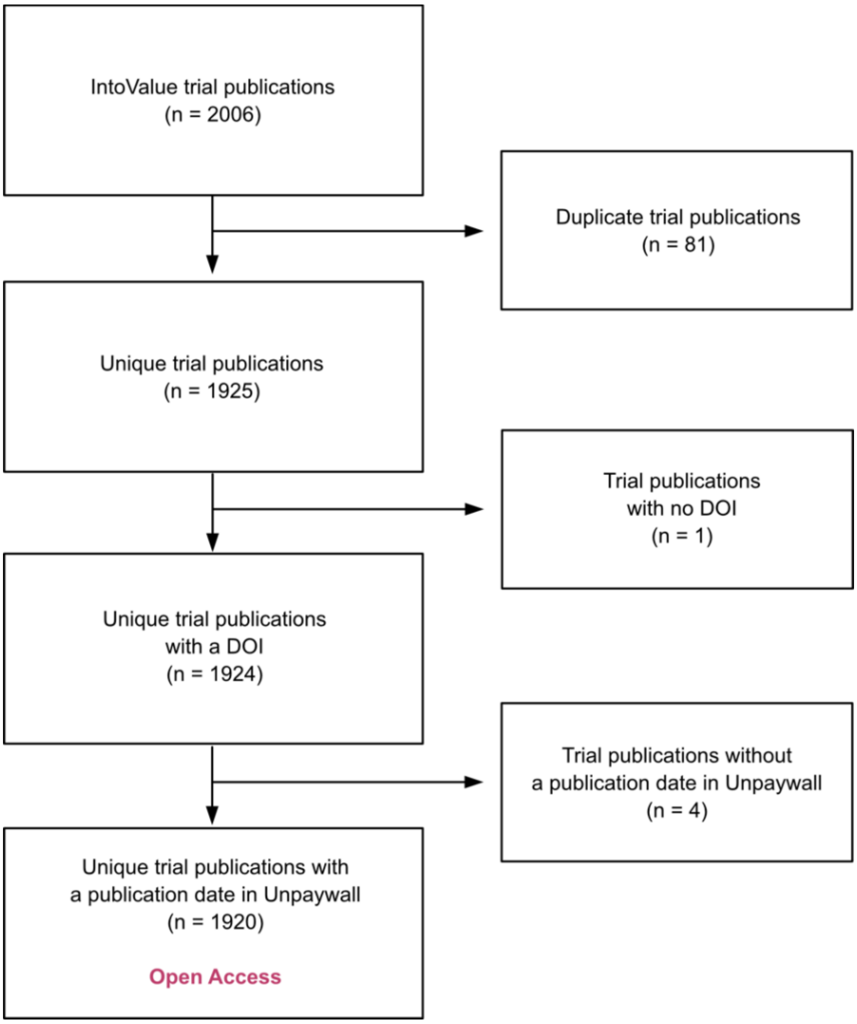

Supplement: S8 Supplement — (PDF) [file pmed.1004175.s008.pdf]
