# Supplementary material for: Institutional dashboards on clinical trial transparency for University Medical Centers: A case study
Source: PLoS Med. 2023 Mar 21;20(3):e1004175. doi: 10.1371/journal.pmed.1004175 (PMC10030018; doi:10.1371/journal.pmed.1004175)
Supplement: S9 Supplement — (PDF) [file pmed.1004175.s009.pdf]

S9 Supplement: Screenshots of the "Start" page of the dashboard

This supplement includes screenshots of select individual panels in the "Start" page of the dashboard (as of 8 November 2022), corresponding to specific results reported in the paper. See the dashboard for an overview of all results.

Prospective registration (ClinicalTrials.gov)

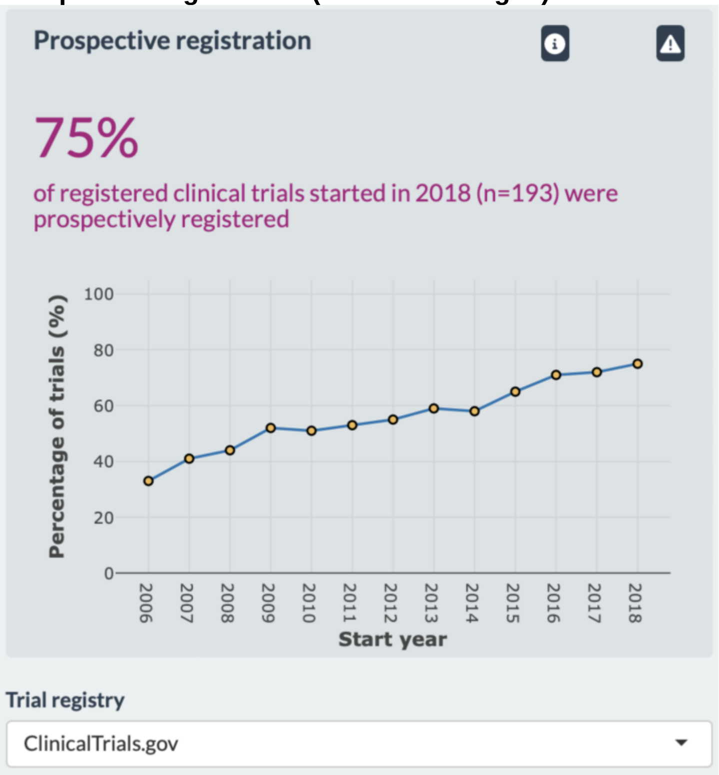

Prospective registration (DRKS)

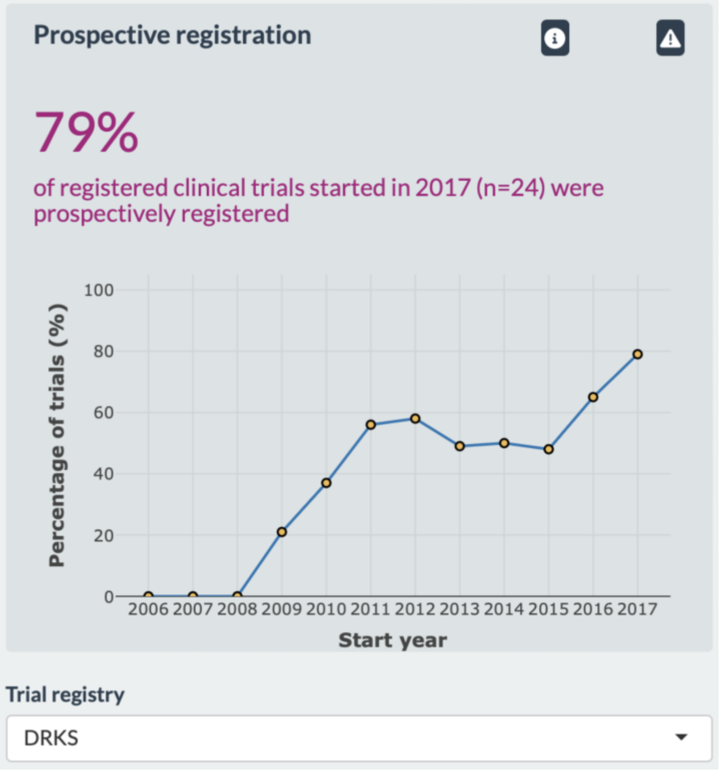

Reporting of a trial registration number in publications

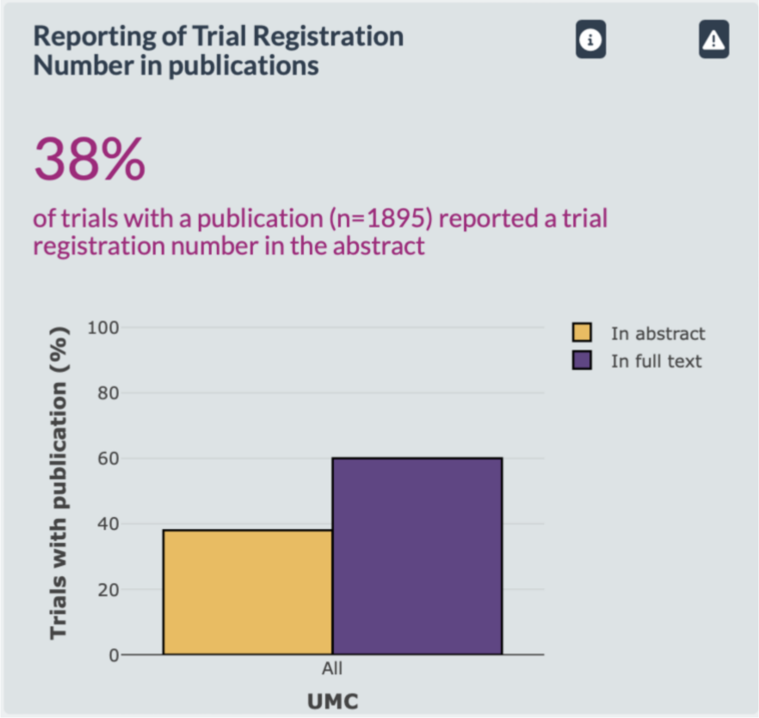

Summary results reporting, cumulative (ClinicalTrials.gov)

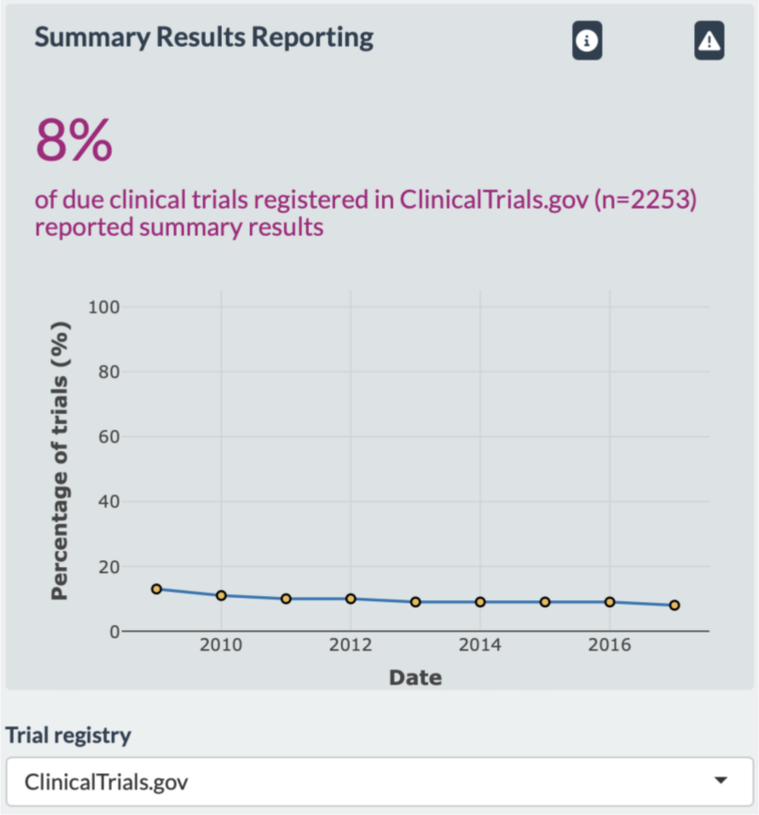

Summary results reporting, cumulative (DRKS)

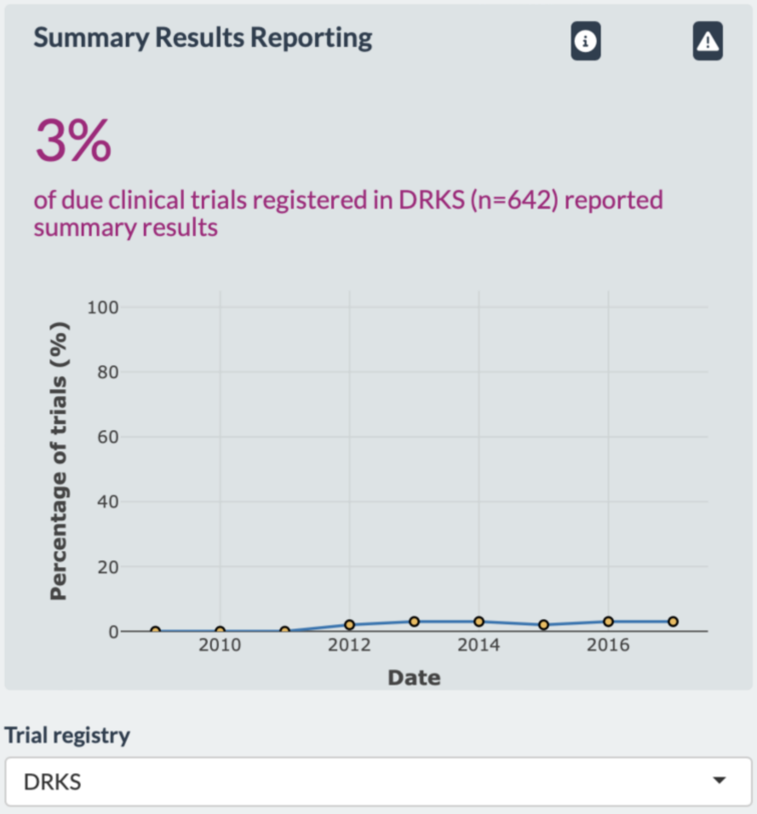

Summary results reporting (EUCTR)

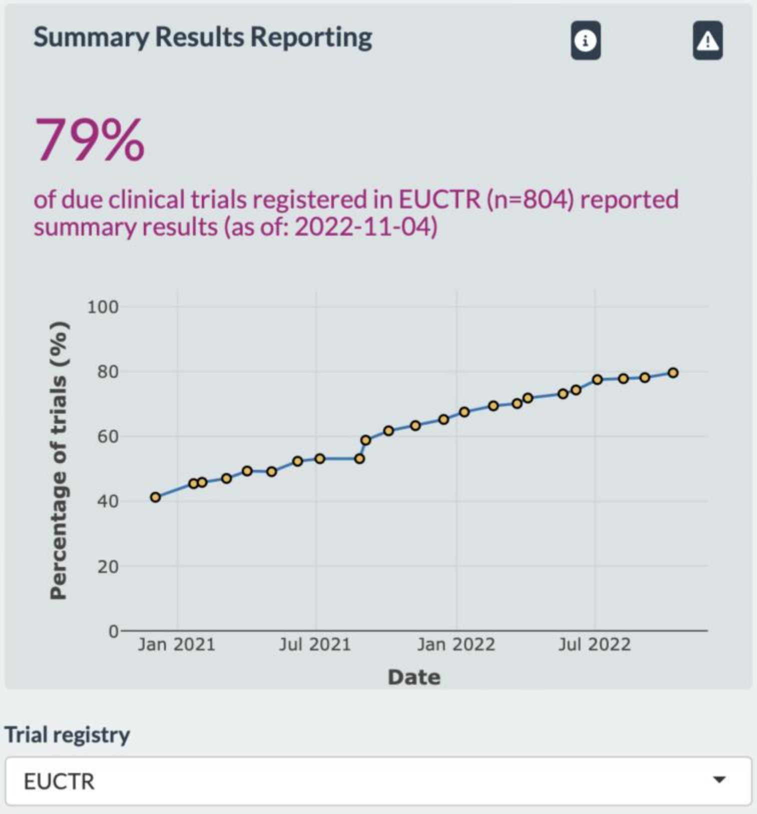

Open Access

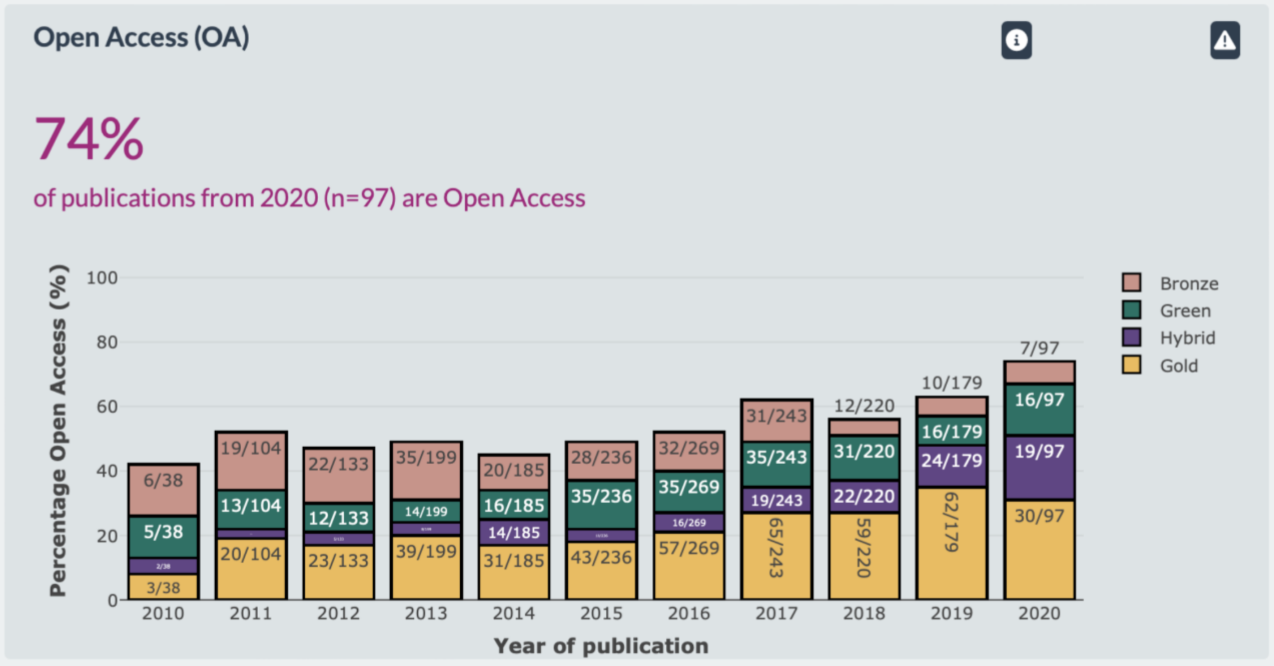
Open Access (OA)
74%
of publications from 2020 (n=97) are Open Access
Percentage Open Access (%)
100
80
60
40
20
0
Bronze
Green
Hybrid
Gold
2010
2011
2012
2013
2014
2015
2016
2017
2018
2019
2020
Year of publication
6/38
5/38
3/38
19/104
13/104
20/104
22/133
12/133
23/133
35/199
14/199
39/199
20/185
16/185
14/185
31/185
28/236
35/236
43/236
32/269
35/269
16/269
57/269
31/243
35/243
19/243
65/243
12/220
31/220
22/220
59/220
10/179
16/179
24/179
62/179
7/97
16/97
19/97
30/97
