# Supplementary material for: Institutional dashboards on clinical trial transparency for University Medical Centers: A case study
Source: PLoS Med. 2023 Mar 21;20(3):e1004175. doi: 10.1371/journal.pmed.1004175 (PMC10030018; doi:10.1371/journal.pmed.1004175)

S10 Supplement: Screenshot of the "One UMC" page of the dashboard

The "One UMC" page of the dashboard displays the assessment of 7 registration and reporting practices for a given UMC contextualized to that across all UMCs (as of 8 November 2022). The example of Charité – Universitätsmedizin Berlin is displayed.

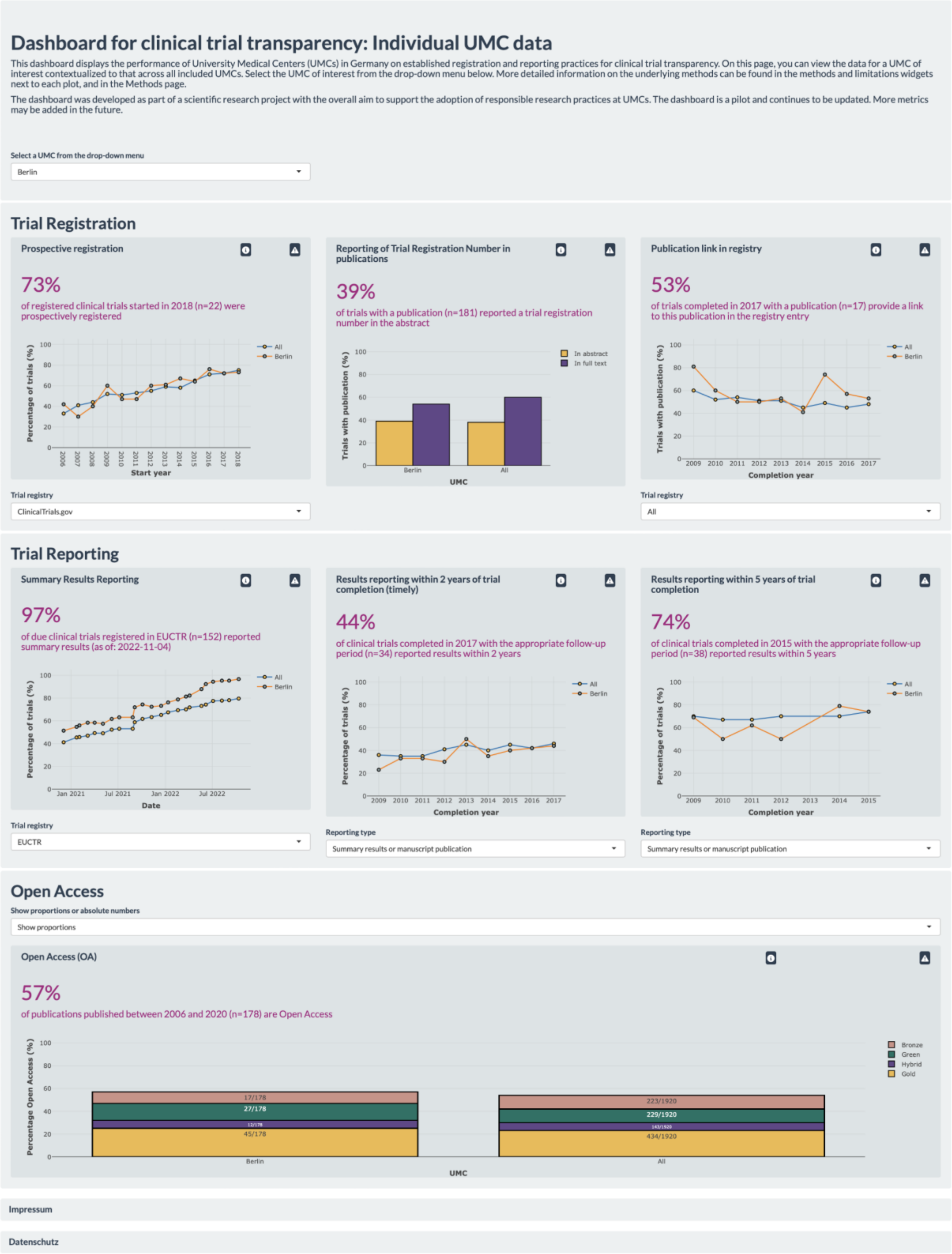

Supplement: S10 Supplement — (PDF) [file pmed.1004175.s010.pdf]
