# Supplementary material for: Institutional dashboards on clinical trial transparency for University Medical Centers: A case study
Source: PLoS Med. 2023 Mar 21;20(3):e1004175. doi: 10.1371/journal.pmed.1004175 (PMC10030018; doi:10.1371/journal.pmed.1004175)
Supplement: Attachment — Submitted filename: response-to-reviewers.docx [file pmed.1004175.s011.docx]

# Response to reviewers

**Manuscript number**: PMEDICINE-D-22-01430R1

We are grateful to the editors and reviewers for the time invested and for their helpful suggestions.

Below we provide a point-by-point response to each of the comments and highlight the corresponding changes we have made to the manuscript in blue text.

We would like to highlight the following changes that are also references in our responses:

·	To ensure the dashboard is as up-to-date as possible, we downloaded updated registry data for this revision. The results in the manuscript have been updated accordingly.

·	The change from 3,790 to 3,113 trials in our base dataset reflects the upfront exclusion of non-German UMC led trials not considered in our study (n=675) and 2 previously missed cross-registrations. We updated the results and flowcharts accordingly. The base trial screening flowchart is now displayed in Fig 2.

·	We included an improved version of the infographic displaying the practices assessed and relevant guidelines and/or laws (Fig 1).

·	We also expanded the Supplementary Materials to provide more information.

## From the Academic Editor:

1. *The authors should pay attention to avoid citation shortcuts. In some occasions, it makes the manuscript difficult to follow and blurs the line between what is new and what was already published. This being said, the paper is very well written, all previous papers are appropriately cited, a web appendix, and online materials are available.*

**Author response: We thank the editor for the positive remarks. We expanded our Supplemental Material to summarize the key methodological information of relevance for this study (Supplements 1-4). The dashboard also summarizes these key points in a Methods page and in widgets besides each plot. We also revised the manuscript with more detailed information on the methods to sample UMC-specific sets of registered clinical trials and associated results: “Briefly, we used automated methods to search registries for clinical trials associated with German UMCs and manually validated the affiliations of all trials. Results publications associated with these trials were identified by means of a manual search across several search engines. This was complemented by automated methods to identify linked publications in the registry.”**

2. *Authors also cite various initiatives (trial tracker, the French Monitor for Open Science, etc). I would be very interested in a detailed comparative assessment of these existing initiatives (in terms of metrics used, timeframe, etc.). Authors cite an initiative about developing a core outcome set. Describing all outcomes used by existing initiatives may inform the development of such a core outcome set.*

**Author response: We very much agree with the editor that a detailed comparative assessment of the increasing number of tools to monitor research outputs and research practices would be valuable and interesting. We consider such a detailed comparison, in addition to the already existing overview, to be beyond the scope of this study and a separate paper in itself. We now highlight this point in the Discussion: “A detailed comparative assessment of existing monitoring initiatives and lessons learned could further support these efforts.”**

3. *The dashboard presented in Figure 2 displays mean values. Of course, this is important. But as this is a dashboard that aims to explore various institutions, one would be interested in values of all institutions (including the max and the min). I wonder if it would be possible to display this information.*

**Author response: We agree and would like to point to the fact that our dashboard visualizes the data in 3 ways, which are difficult to display in the manuscript: 1) Performance across all UMCs (“Start” page), 2) Performance of all UMCs side-by-side (“All UMCs” page”), and 3) Performance of a single UMC compared to that across all UMCs (“One UMC” page, the UMC of choice can be selected in a drop-down menu). The “All UMC” page in particular addresses this point by showing the minimum and maximum across all institutions.**

4. *One major problem of these monitors is that they are based on data which are likely biased. I'm not sure that trials registries are appropriately updated by all UMCs, e.g. regarding study status. It may introduce a lot of bias in the output, and this bias could be differential when one compares UMCs. This possible issue should be discussed in more detail as it is a major limitation of this comparative approach.*

**Author response: The editor raises the important point that parts of the dashboard rely on the data in trial registries (ClinicalTrials.gov and DRKS) being accurate and up-to-date. Transparency practices were assessed for a cohort of interventional clinical trials that were reported as complete between 2009 and 2017 on the trial registry at the time of data collection. This was based on the completion date in AACT (ClinicalTrials.gov) and the study end date in the German Clinical Trials Register (DRKS). While this information should be kept up-to-date in the registry, we recognize that this is not always the case (e.g., an anticipated completion date may not have been updated to reflect the actual date of trial completion; trial status may not be up-to-date). Nevertheless, trial registries remain the most comprehensive source of information on planned, ongoing, and completed clinical trials. Moreover, the changes in completion dates that we found for our dataset did not reflect systemic biases toward any institution over another per se.**

**We address this limitation in 4 ways:**

- **Recognizing that registry data is imperfect and subject to change and corrections over time, we attempted to reflect the most accurate status of clinical trials. We therefore updated the registry data with the most recent data we could obtain (query date: 1 November 2022). Of trials led by a German UMC, this resulted in the exclusion of an additional 8 trials (73 instead of 65) based on completion date and 9 trials (16 instead of 7) based on status; this represents 0.5% of our sample (n=2895 trials). Furthermore, a preliminary check of trials that no longer met the inclusion criterion “completion date 2009 - 2017” revealed no substantial bias towards specific UMCs. We plan to refresh the dashboard to newer registry data in an ongoing manner. We have updated the Results and flowcharts to reflect the updated registry data.**
- **We surveyed trialists responsible for the trials in this dataset in the context of two related projects (manuscripts in preparation, see** [**https://osf.io/stnp5/**](https://osf.io/stnp5/) **for preregistration of one project). We invited trialists to flag any corrections to the trial data; this led to only minimal changes to the number of publications and no change in the overall conclusion.**
- **The dashboard transparently communicates the limitations of the data sources, including registries: each plot includes widgets on limitations, and the Methods page presents limitations across all metrics and data sources.**
- **We have now explicitly added this point in the:**
  - **Abstract: “A limitation of this study is that some of the methods used to assess the transparency practices in this dashboard rely on registry data being accurate and up-to-date.”**
  - **Discussion: “A limitation for this study is that inaccurate or outdated registry data (e.g., incorrect completion dates or trial status) may have impacted the assessment of transparency practices described in this study. To mitigate this limitation, we updated the registry data with the most recent data we could obtain. The update-related changes suggest no systematic bias in the comparison across UMCs.”**

5. *Authors may want to discuss their results in line with those of Speich et al. PLOS Medicine. 2022 and Thiele et al. Plos One 2022 ;*

**Author response: We now refer to both references in the Discussion: “Beyond transparency, other potential metrics for a dashboard could reflect the number of discontinued trials [53] or the proportion of trials that inform clinical practice [54]. The development of such metrics should acknowledge the availability of standards and infrastructure pertaining to the underlying practices [23] and differences between study types and disciplines [27]. Future versions of the dashboard may also display additional sub-population comparisons, such as different clinical trial registries or UMC particularities [55].”**

*6. Introduction:*

*– I would stress that there is a very favorable environment for these initiatives*

*–More emphasis on DORA and the Hong Kong principles is needed ;*

*– I would also welcome more emphasis on the European approach for rewarding reproducible research practices and Open Science ;*

*–I would also cite the UNESCO approach of Open Science ;*

**Author response: We thank the editor for this suggestion and have revised the Introduction accordingly. Our revision reads: “Initiatives such as the Declaration on Research Assessment (DORA) and the Hong Kong Principles have called for a change in the way researchers are assessed, and for more recognition of behaviors that strengthen research integrity [20,27]. The European Commission’s Agreement on Reforming Research Assessment emphasizes this shift towards rewarding responsible research practices to maximize research quality and impact [28]. In turn, the UNESCO Recommendation on Open Science adopted in 2021 affirmed the need to establish monitoring and evaluation mechanisms relating to open science [29].”**

*7. Methods:*

*– I would provide more details on the automatic versus manual part of the extraction as this probably a very big problem for reproducibility regarding data extraction ;*

*–A citation is needed concerning the list of German UMC that was used ;*

*.--p6. 115, a citation is needed after has been described previously ;*

*–Again, the interactive dashboard is the most important and original part and it needs more emphasis ;*

**Author response: We have addressed these comments as follows:**

- **We added a Supplement with an overview of the steps involved in creating the dashboard, and their dependency on automated vs. manual approaches (see Supplement S1)**
- **We clarified how we obtained the list of German UMCs to include in the Methods, and provided a citation to the association of medical faculties in Germany (Medizinischer Fakultätentag): “Trials were searched for 38 German UMCs based on their inclusion as members on the website of the association of medical faculties [36] at the time of data collection.”**
- **We added a citation after “has been described previously”**
- **To emphasize the dashboard, we revised the Results as follows: “The key outcome of this paper is an interactive and openly accessible dashboard to visualize adherence to the aforementioned best practices for trial registration and reporting across German UMCs: https://quest-cttd.bihealth.org/.”**

*8. Results:*

*–The various flow charts in the supplements are very informative but difficult to follow. I was wondering if a single flow chart with various branches would be a better figure (I acknowledge that this part is very challenging to represent adequately) ;*

**Author response: We appreciate the editor’s comment and initially tried to display this information in a single flowchart. However, given the different denominator for each practice, it was very challenging to represent them all to fit within a journal page. Therefore, we opted for this step-wise approach with a separate flowchart for each set of practices that share the same denominator. In response to this feedback, we separated the flowchart for the baseline denominator from which most of the registry-based practices stem (n = 2,895). See Figure 2.**

*9. Discussion:*

*– Author should elaborate a little bit more on the frequency of updates etc.*

**Author response: We have added more information on the frequency of planned updates in the Discussion: “We are planning regular updates of the registry data for trials already in the dashboard, as well as the inclusion of more recent cohorts of trials with at least 2 years follow-up (e.g., trials completed 2018-2021 assessed in 2023).”**

*–Authors should also discuss the implication of this dashboard, for instance, should the trial registry implement such a dashboard directly? Which international organisation may be interested in using at a large scale such a dashboard? How difficult would it be to transfer such a dashboard from Germany to the rest of the world?*

**Author response: We thank the editor for this comment. Based on preliminary conversations with different stakeholder groups in Germany and abroad, we are very optimistic about interest in and implementation of this dashboard approach. For example, German clinical trial funders expressed their interest in implementing such a dashboard to monitor compliance with their policies as well as applicable guidelines and laws. We are currently preparing such a project together with relevant organizations. We are aware of similar efforts at the level of registries, and are in communication with leadership at the ISRCTN registry with regards to their transparency tracker (**[**https://www.isrctn.com/search/tracker**](https://www.isrctn.com/search/tracker)**). In terms of transferring such a dashboard beyond Germany: the code to create the dashboard is openly available and can be adapted to other cohorts. The generation of the underlying dataset of clinical trials and associated results publications involves resource-intensive manual checks. In turn, the assessment of transparency practices involves mainly automated methods. All these methods are openly available and are described in previous publications cited in the manuscript. We added a Supplemental file that provides an overview of the steps of our approach and their dependency on automated vs. manual methods. We have already successfully expanded some of our activities with collaborators beyond Germany, for example in the context of an assessment of clinical trial transparency at select medical schools in California (protocol:** [**https://osf.io/u9d5c/**](https://osf.io/u9d5c/)**) as well as across Nordic countries.**

**Clinical trial offices have also expressed interest in such a dashboard and related communication strategies, which we have presented to the German Association of Medical Faculties as well as the US Clinical Trials Registration and Results Reporting Taskforce. We revised the Discussion with the following: “The datasets and methods used in this study can be scaled: this has been demonstrated in another European country (Poland) [49] and is currently underway in California, USA [50]. While the generation of the underlying dataset of clinical trials and associated results publications involves manual checks (approximately 10 person-hours per 100 trials), the assessment of transparency practices is largely automated. Institutions in possession of an in-house cohort of clinical trials registry numbers and persistent identifiers (e.g., DOIs) from matched journal publications, however, could achieve results more quickly. The code to create the dashboard is openly available and can be adapted to other cohorts.”**

*–Authors should stress that implementation of such dashboard will not guarantee that UMCs will be more transparent. What kind of research could be envisioned to answer this question? I would love to see more elaboration on this point.*

**Author response: We agree this is another important point. The main objective of this dashboard is to communicate trial transparency assessments with UMCs to support their efforts to improve. While the dashboard does not in itself guarantee that UMCs will be more transparent, it provides a baseline against which future changes can be measured. To evaluate change in UMC transparency, we recently conducted an intervention at the Charité - Universitätsmedizin Berlin to evaluate the impact of tailored trial transparency “report cards” with feedback and guidance to increase clinical trial transparency (protocol:** [**https://osf.io/stnp5/**](https://osf.io/stnp5/)**). Publications describing the report cards approach and the results of the pilot study at the Charité - Berlin are in preparation.**

**We now address this point in the Discussion: “UMCs and their core facilities for clinical research can, for example, use the data linked to the dashboard to inform principal investigators about the transparency of their specific trials. We are currently finalizing such a “report card” approach at the Charité - Universitätsmedizin Berlin [48].”**

**We further revised the section in the discussion addressing stakeholder and community engagement. The relevant text is: “The uptake of this dashboard approach by UMCs and other stakeholders depends on their respective attitudes and readiness. We previously solicited stakeholders’ views on an institutional dashboard with metrics for responsible research. While interviewees considered the dashboard to be helpful to see where an institution stands and to initiate change, some pointed to the challenge that making such a dashboard public might risk incorrect interpretation of the metrics and harm UMCs’ reputation [40]. While similar challenges with interpretation and reputation apply to current metrics for research assessment (e.g., impact factors and third-party funding), this stakeholder feedback demonstrates the need for community engagement when introducing novel strategies for research assessment. In this regard, a Delphi study was performed to reach consensus on a core outcome set of open science practices within biomedicine to support audits at the institutional level [51].”**

### *Other editorial points:*

*10. Title: Please revise your title according to PLOS Medicine's style. Your title must be nondeclarative and not a question. It should begin with main concept if possible. "Effect of" should be used only if causality can be inferred, i.e., for an RCT. Please place the study design ("A randomized controlled trial," "A retrospective study," "A modelling study," etc.) in the subtitle (ie, after a colon).*

**Author response: Based on these recommendations, we have changed the title to: “Institutional dashboards on clinical trial transparency: A case study for University Medical Centers.”**

*11. Data availability statement: Please also include a link to the dashboard, where it will be made publicly available.*

**Author response: We have added a link to the dashboard in the data availability statement.**

*12. Abstract: Please combine the Methods and Findings sections into one section, “Methods and findings”.*

**Author response: We have combined these sections in the Abstract.**

*13. Abstract: Line 11: Please define DRKS at first use.*

**Author response: We have now defined DRKS in this particular instance.**

*14. Abstract: Methods and Findings: Please describe how adherence to established transparency practices was assessed.*

**Author response: We have added the following sentence in the Abstract to describe how adherence to established transparency practices was assessed: “To assess adherence to transparency practices, we identified results publications associated to trials and applied automated methods at the level of registry data (e.g., prospective registration) and publications (e.g., open access).”**

15. Abstract: Methods and Findings: Please ensure that all numbers presented in the abstract are present and identical to numbers presented in the main manuscript text.

**Author response: We thank the editor for this comment. The numbers were not identical in all cases, as we provided overall numbers in the Abstract but more specific trends in the Results. We have now ensured that the numbers in Abstract can also be found in the Results section.**

16. Abstract: Methods and Findings: In the last sentence of the Abstract Methods and Findings section, please describe the main limitation(s) of the study's methodology.

**Author response: We have added the main limitation in the last sentence of the Abstract Methods and Findings section: “A limitation of this study is that some of the methods used to assess the transparency practices in this dashboard rely on registry data being accurate and up-to-date.”**

17. Abstract: Conclusions: * Please address the study implications without overreaching what can be concluded from the data; the phrase "In this study, we observed ..." may be useful.

**Author response: We have addressed this comment by revising the first sentence of the Conclusions section of the Abstract as follows: “In this study, we observed that it is feasible to assess and inform individual UMCs on their performance on clinical trial transparency in a reproducible and publicly accessible way.”**

*18. Author summary: At this stage, we ask that you include a short, non-technical Author Summary of your research to make findings accessible to a wide audience that includes both scientists and non-scientists. The Author Summary should immediately follow the Abstract in your revised manuscript. This text is subject to editorial change and should be distinct from the scientific abstract. Please see our author guidelines for more information:* [*https://journals.plos.org/plosmedicine/s/revising-your-manuscript#loc-author-summary*](https://journals.plos.org/plosmedicine/s/revising-your-manuscript#loc-author-summary)

**Author response: We have drafted a non-technical author summary of the research below the Abstract.**

*19. Main text: Please place in-text citations for references within square brackets. Where multiple references are indicated, please do not include spaces within brackets.*

**Author response: Done.**

*20. Main text: Please define all acronyms at first use in the text.*

**Author response: We thank the editor for this comment. We have checked this throughout the manuscript and defined all acronyms at first use in the text.**

*21. Methods: For all observational studies, in the manuscript text, please indicate: (1) the specific hypotheses you intended to test, (2) the analytical methods by which you planned to test them, (3) the analyses you actually performed, and (4) when reported analyses differ from those that were planned, transparent explanations for differences that affect the reliability of the study's results. If a reported analysis was performed based on an interesting but unanticipated pattern in the data, please be clear that the analysis was data-driven.*

**Author response: The aim of this study was to develop an institutional dashboard to communicate trial transparency assessments to German UMCs. We generated descriptive statistics on the characteristics of the trials and the transparency practices across UMCs. We did not test specific hypotheses. We added a section in the Methods entitled ‘Analysis’ which reads: “We generated descriptive statistics on the characteristics of the trials and the transparency practices, all of which are displayed in the dashboard. We report proportions across UMCs (e.g., “Start” page) and per UMC (e.g., “One UMC”) broken down by start year (prospective registration only), completion year, registry (publication linkage in registry, summary results reporting), and publication year (open access). We did not test specific hypotheses.”**

*22. Methods: Did your study have a prospective protocol or analysis plan? Please state this (either way) early in the Methods section.*

*a) If a prospective analysis plan (from your funding proposal, IRB or other ethics committee submission, study protocol, or other planning document written before analyzing the data) was used in designing the study, please include the relevant prospectively written document with your revised manuscript as a Supporting Information file to be published alongside your study, and cite it in the Methods section. A legend for this file should be included at the end of your manuscript.*

*b) If no such document exists, please make sure that the Methods section transparently describes when analyses were planned, and when/why any data-driven changes to analyses took place.*

*c) In either case, changes in the analysis-- including those made in response to peer review comments-- should be identified as such in the Methods section of the paper, with rationale.*

**Author response: The development of the dashboard was not preregistered and there was no protocol or analysis plan. However, we would like to highlight that the methods to develop the underlying dataset of clinical trials and associated results publications were pre-registered in OSF (IntoValue 1:** [**https://osf.io/fh426/**](https://osf.io/fh426/)**, IntoValue 2:** [**https://osf.io/98j7u/**](https://osf.io/98j7u/)**). These are already linked in the respective publications. We now explicitly state this in the Methods section, as follows: “The development of the dashboard was iterative and did not have a prospective protocol. The methods to develop the underlying dataset of clinical trials and associated results publications, however, were preregistered in Open Science Framework (OSF) for trials completed 2009-2013 [33] and 2014-2017 [34].”**

*23. Methods: Please report your study according to the relevant guideline, which can be found here:* [*http://www.equator-network.org/*](http://www.equator-network.org/)

*Please ensure that the study is reported according to the STROBE or other guideline, and include the completed STROBE (or other) checklist as Supporting Information. When completing the checklist, please use section and paragraph numbers, rather than page numbers. Please add the following statement, or similar, to the Methods: "This study is reported as per the Strengthening the Reporting of Observational Studies in Epidemiology (STROBE) guideline (S1 Checklist)." (Or please adapt to the most appropriate guideline for your study.)*

**Author response: We have completed the STROBE guideline for cross-sectional studies and have added the suggested sentence in the Methods.**

*24. Methods Line 93-95: Although described elsewhere, it would be helpful to have the methods for sampling and tracking results described here.*

**Author response: We have added more information on the methods for sampling and tracking results in this section: “Briefly, we used automated methods to search registries for clinical trials associated with German UMCs and manually validated the affiliations of all trials. Results publications associated with these trials were identified by means of a manual search across several search engines. This was complemented by automated methods to identify linked publications in the registry.”**

*25. Results: Please present numerators and denominators for percentages.*

**Author response: We have checked the Results and have made sure percentages are always accompanied by numerators and denominators.**

*26. Discussion: Please present and organize the Discussion as follows: a short, clear summary of the article's findings; what the study adds to existing research and where and why the results may differ from previous research; strengths and limitations of the study; implications and next steps for research, clinical practice, and/or public policy; one-paragraph conclusion.*

**Author response: We substantially reorganized the Discussion to capture the many related comments from the editor and the four reviewers. We tried but found it impossible to address these different aspects under some of the titles suggested by the editor. In the revised version, we start as suggested with a short summary, and we end with the limitations and a one-paragraph conclusion. We introduced the following subtitles to the Discussion section to capture the several recommendations for the Discussion from the editor and the reviewers:**

- **Trends in trial transparency**
- **Actionable areas for stakeholders**
- **Scalability beyond German UMCs**
- **Stakeholder and community engagement**
- **Updates and further development of the dashboard**
- **Limitations**
- **Conclusions**

**If further reorganization of the Discussion is necessary, we will revise the text again to meet this requirement.**

*27. Lines 336-349: Please remove the Funding, Author contributions, and Competing Interests sections from the main text. Please be sure all information is completely and accurately entered into the manuscript submission system.*

**Author response: We have removed the Funding, Author contributions, and Competing Interests.**

*28. References: Please use the "Vancouver" style for reference formatting, and see our website for other reference guidelines* [*https://journals.plos.org/plosmedicine/s/submission-guidelines#loc-references*](https://journals.plos.org/plosmedicine/s/submission-guidelines#loc-references)

**Author response: We use the Vancouver style for reference formatting.**

*29. Table 1: In the legend, please define all abbreviations used in the table.*

*30. Supplement S2: In the legend, please define all abbreviations used in the table.*

*31. Supplement S4: In the legend, please define all abbreviations used in the table.*

*32. Supplement S5: Please define all abbreviations used in the flowcharts.*

**Author response for comments 29-32: We have defined all abbreviations used in Table 1, Supplements S2, S4, and S5.**

# Comments from the reviewers:

## Reviewer #1:

1. *Please report p-values and 95% confidence interval in the abstract, and results section*

**Author response: We thank the reviewer for this comment. The aim of this study was to develop an institutional dashboard to communicate trial transparency assessments to German UMCs. We provide descriptive statistics on transparency practices across and between UMCs. We now report 95% confidence intervals in the abstract and results section. We did not intend to test specific hypotheses, therefore we did not provide p-values. We added a section in the Methods entitled ‘Analysis’ where we provide more information on the analytical methods used in this study: “We generated descriptive statistics on the characteristics of the trials and the transparency practices, all of which are displayed in the dashboard. We report proportions across UMCs (e.g., “Start” page) and per UMC (e.g., “One UMC”) broken down by start year (prospective registration only), completion year, registry (publication linkage in registry, summary results reporting), and publication year (open access). We did not test specific hypotheses.”**

2. *Line 162/165: I suggesting adding more details about why 3790=2915=875 studies were excluded*

**Author response: The dashboard is based on the IntoValue 1 & 2 cohorts of registered clinical trials and associated results publications (**[**https://zenodo.org/record/5141343#.Y2PeSi8w1N0**](https://zenodo.org/record/5141343#.Y2PeSi8w1N0)**) (n = 3,790 trials). The IntoValue 1 cohort included 675 trials where a German UMC is a facility contributor (rather than a sponsor, responsible party, or host of the principal investigator). Given our focus on trials led by German UMCs, we excluded these German UMC facility trials from the base dataset, i.e., before the trial screening. We further excluded 2 trials that were missed cross-registrations in ClinicalTrials.gov/DRKS (see more details in response #2 to Reviewer #3). As a result, the starting dataset now consists of 3,113 trials.**

**In an effort to display the most up-to-date trial data, we downloaded updated registry data for all trials (query date: 1 November 2022). We then re-applied our exclusion criteria: not complete per study status in the registry, not completed 2009 - 2017, not interventional. This led to the exclusion of 91 trials. After exclusion of duplicate trials between IntoValue 1 & 2 (n=127), the dataset consisted of 2,895 trials. This dataset served as the basis for most of the practices displayed in the dashboard (see flowcharts in Supplement 8 for more details). To address this comment, we provide the trial screening flowchart as a Figure in the main text (Fig 2). We also revised the Results section “Characteristics of trials” as follows: “The IntoValue dataset that this study is based on includes interventional trials registered in ClinicalTrials.gov or DRKS, led by a German UMC, and completed between 2009 and 2017 (n = 3,113). Trials were found for 35 out of 38 UMCs searched. After downloading updated registry data for trials in this cohort, we excluded 91 trials based on our exclusion criteria (not completed 2009 – 2017, n = 73; not complete per study status, n = 16; not interventional, n = 2). After removal of duplicates, this led to 2,895 trials which served as the basis for most metrics (Fig 2).”**

3. *The analysis mainly focused on the description of the overall "clinical transparency" over time. What are the underlying reasons for the changes overtime? Is it possible to examine the heterogeneity in the outcomes in study characteristics(e.g. individual UMC, size of study sample, disease category, results of the trial [ effective or not])*

**Author response: We thank the reviewer for this comment and agree that this is an important question to address. We would like to point out that both earlier studies that generated the cohort of trials and publications underlying this dashboard performed exploratory logistic regressions to identify variables that may have an influence on the timely publication of results. These regression analyses are already published in the two respective publications that are also referenced in the present paper. For more details on the regression analysis see, for example,** [**https://osf.io/m4brf**](https://osf.io/m4brf)**. We agree that it would be interesting to perform regression analyses for further practices beyond timely publication of results. However, we consider these analyses beyond the scope of this manuscript which has the primary objective of presenting a dashboard to communicate transparency assessments to UMCs. We would also like to point out that the dashboard allows the data to be explored in different ways (e.g., filter by registry), enabling users to explore heterogeneity in the outcomes in study characteristics. The access credentials for the dashboard are now available directly in the manuscript.**

## Reviewer #2:

*I would like to congratulate the authors on a thorough review of trial registration and results posting in the German Academic research environment. Although we could not see it completely, I also applaud the creation of a Dashboard for future data display. I have a few comments for the authors' consideration.*

**Author response: We thank the reviewer for this feedback. We are sorry to hear that the dashboard was not accessible. We now provide the credentials to access the dashboard directly in the manuscript. The dashboard will be fully publicly available upon publication in a journal.**

1. *The authors need to carefully distinguish disclosure (which is what they discuss) from transparency. Disclosure is a necessary but not sufficient step in transparency and open science. As the authors point out the disclosures need to findable and accessible to meet the FAIR principles. It would be good for the authors to reference FAIR upfront as they underpin open science in general. You have clearly outlined the principles on page 3 line 38.*

**Author response: We thank the reviewer for this comment. We revised the Introduction to distinguish disclosure from transparency and integrated recognition of FAIR by citing the FAIR Principles. The revised sentence reads: “For clinical trials to generate useful and generalizable medical knowledge gain, trial results should also be reported in a timely manner after trial completion per the World Health Organization Joint Statement on Public Disclosure of Results from Clinical Trials [2]. Disclosure is a necessary but not sufficient component of transparency: trial results should also be openly accessible and findable, in line with established guidelines [2–6].”**

2. *There needs to be care in comparing results to clinical trials.gov. Note that the requirement to register and post results does not apply to Phase I trials and that might or might not affect the denominator. Also, before 2017 the results requirements only applied to trials of approved medicines so again the denominator for posted results may not be ALL trials. It is true that the ICMJE criteria were broader than that and while of great influence were not strictly speaking legal requirements.*

**Author response: We thank the reviewer for raising this important point. In this and related studies, we used the WHO definition of a clinical trial, i.e., “any research study that prospectively assigns human participants or groups of humans to one or more health-related interventions to evaluate the effects on health outcomes” (**[**https://www.who.int/clinical-trials-registry-platform/about/glossary**](https://www.who.int/clinical-trials-registry-platform/about/glossary)**). Trials displayed in the dashboard include all studies that also meet our inclusion criteria (interventional, registered in ClinicalTrials.gov or DRKS, led by a German UMC, considered as complete between 2009 and 2017 per the registry). Thus, this dataset is not limited to (but may include) Clinical Trials of an Investigational Medicinal Product (CTIMPs) regulated by laws. We are only assessing compliance with legal requirements for summary results reporting in EudraCT, which we obtain from the EU Trials Tracker (see first plot under “Trial Reporting” in the start page of the dashboard). Where a trial is not regulated by law, trial sponsors still have a moral obligation to register clinical trials prospectively (Declaration of Helsinki) and the trial’s results should be reported in line with established guidelines (e.g., WHO, ICMJE, CONSORT). The dashboard evaluated adherence to these guidelines. We have revised the Methods to highlight this point: “In this study, we focused on adherence to ethical principles and reporting guidelines that apply to all trials. Compliance with a legal regulation was only assessed for summary results reporting in the EUCTR.”**

3. *P 3 line 40 You refer to "registered" with reference to results- I assume you mean there are results posted for trials that were not registered?*

**Author response: We meant to point to the fact that clinical trials are often not registered or reported in accordance with established guidelines (i.e., retrospectively registered, not reported in a timely manner, not findable, or not accessible). Because the text points to these standards, we have revised this sentence to: “However, several studies have shown that clinical trials are often not registered and reported according to these standards.”**

4. *P 5 - It would be useful to define what you mean by "prospective". For CT.Gov the requirement is to register no later than 21 days after the first patient is enrolled. Is that the definition you are using?*

**Author response: We acknowledge the reviewer’s comment that the evaluation of “prospective registration” may be implemented differently. We would like to point the reviewer to Supplement S5, where we define how prospective registration in ClinicalTrials.gov and DRKS was defined in this study: “To assess whether a study was prospectively registered, we compared the date the study was first submitted to the registry with the start date given in the registry. We defined a trial to be prospectively registered if the trial was registered in the same or a previous month to the trial start date, as some registrations provide only a start month rather than an exact date.”**

5. *P 8 line 163. Under results when you use the term "registered" are you using the "prospective" definition or all? In other places you are specific.*

**Author response: When we use the term “registered”, we mean all registered trials (prospective and retrospective). The dataset the dashboard is based on only includes clinical trials that were registered in ClinicalTrials.gov or DRKS (German Clinical Trials Register). We revised the text to make this point clearer in the Methods: “The data displayed in the dashboard relate exclusively to registered (either prospectively or retrospectively) clinical trials obtained from three data sources with the following inclusion and exclusion criteria”.**

6. *P 9 line 192: I agree part of findable means referring to the TRN. It is disappointing journals are not enforcing that and perhaps that needs to called out.*

**Author response: We agree with the reviewer and would like to point to a previous publication from our research group. We now address this in the Discussion and point to a previous paper from our group which calls on journals to enforce TRN reporting: “Some of the practices included in this dashboard can still be addressed retrospectively, such as linking publications in the trial registration (realized for 49% of trials with a publication). These constitute actionable areas for improvement that UMCs can contribute to by providing education, support, and incentives. One important way to incentivize UMCs in this regard is to make responsible research practices part of internal and external quality assessment procedures. Other stakeholders such as funders, journals and publishers, registries, and bibliographic databases should complement these activities by reviewing compliance with their policies as well as applicable guidelines and/or laws (see recent activities at the ISRCTN: https://www.isrctn.com/search/tracker). Salholz-Hillel et al. (2022), for example, outlined specific recommendations for each stakeholder to improve links between trial registrations and publications [10].”**

7. *In the discussion you mention for the first time in the paper preclinical research and while the statement may be true you have not addressed that anywhere else and I suggest you might delete that here as it is a new concept. You also refer to "clinical research" in reference to your dashboard, but it is really clinical trials, correct? There are many discussions surrounding the registration of observation studies and I assume your dashboard is restricted to interventional trials as noted in the beginning?*

**Author response: We thank the reviewer for these helpful comments. We have removed the mention of preclinical research in the discussion. The reviewer is correct that the dashboard is restricted to interventional clinical trials. We acknowledge this point and have changed the relevant instances of “clinical research” to specify “clinical trials” in the manuscript and dashboard. The sentence in question in the Discussion now reads: “Concerns about delayed and incomplete results reporting in clinical research and other sources of research waste have triggered debate on incentivizing individual researchers and UMCs to adopt more responsible research practices”.**

## Reviewer #3:

1. *The authors describe the development of a dashboard to illuminate execution of commitments to transparency in clinical trials. Note that many of the substantive results have been published previously, including trial registration, results reporting of trials, linkage of publications to trial registration and others. Therefore, the description and utility of the dashboard is the substantive new contribution, but it is rather briefly described (e.g., one paragraph in the manuscript). The description, development, user engagement, potential adaptations etc should be more fully described.*

**Author response: We believe that this comment should be partially addressed by having access to the dashboard (we are sorry to hear that this was not the case). We now provide the access credentials directly in the manuscript. The dashboard will be fully publicly available upon publication in a journal. The development of the dashboard was informed by stakeholder interviews on a prototype institutional dashboard with metrics for responsible research (**[**https://journals.plos.org/plosone/article?id=10.1371/journal.pone.0269492**](https://journals.plos.org/plosone/article?id=10.1371/journal.pone.0269492)**). We also provide more information on the dashboard development in the Methods: “The dashboard was shaped by interviews with UMC leadership, support staff, funders, and experts in responsible research who provided feedback on a prototype version [40]. This feedback led to the inclusion of several features to facilitate the interpretation of the data and contextualize the practices included.”**

2. *It would be very important to deduplicate the trials that are reported both in Clinicaltrials.gov and DRKS and/or divide the results by those that are registered and reported in one but not the other or both. The expectations and nature of the trials may differ.*

**Author response: We thank the reviewer for raising this important point. We deduplicated trials cross-registered in both ClinicalTrials.gov and DRKS. If the DRKS registration mentioned a ClinicalTrials.gov identifier as a secondary ID, we favored the registration in ClinicalTrials.gov:** [**https://github.com/quest-bih/IntoValue2/blob/master/code/1_sample_generation/Create_DRKS_sample.R#L110**](https://github.com/quest-bih/IntoValue2/blob/master/code/1_sample_generation/Create_DRKS_sample.R#L110) **and** [**https://osf.io/d4mes**](https://osf.io/d4mes)**. We have now specified this in the Methods: “We deduplicated trials cross-registered in both ClinicalTrials.gov and DRKS (see more information in Supplement S2).”**

**Supplement S4 includes a table of characteristics of included trials divided between ClinicalTrials.gov and DRKS. We recognize that there could be additional cross-registrations that were missed in the automated check. In the context of a related project on a subset of these trials (n = 168), we manually checked all potential cross-registrations based on 1) ClinicalTrials.gov identifier in DRKS, 2) DRKS identifier in ClinicalTrials.gov, or if applicable, 3) either ClinicalTrials.gov or DRKS identifier in the results publication. See the protocol:** [**https://osf.io/mjpzh**](https://osf.io/mjpzh)**. We found 2 missed cross-registrations, accounting for 1% of the sample. We updated the sample used in the dashboard to remove these 2 trials. Based on this, we therefore believe missed cross-registrations to represent a very small portion of our sample. We now address this in the limitations: “Another limitation is that the trial dataset may contain more cross-registrations than we identified. For the aforementioned “report card” project, we manually verified 168 trials and found only 2 missed cross-registrations (1%). We therefore believe that missed cross-registrations represent only a small portion of our sample.”**

3. *Did the authors analyze by type of trial (regulated, drug/biologic/device/behavioral, industry v other, etc.) differences in registration, results reporting, linkage of registration to publications, and the other outcome measures? If not, why not?*

**Author response: We thank the reviewer for this comment. We agree that this is an important question to address. We would like to point out that both earlier studies that generated the cohort of trials and publications underlying this dashboard performed exploratory logistic regressions to identify variables that may have an influence on the timely publication of results. These regression analyses are already published in the two respective publications that are also referenced in the present paper. For more details on the regression analysis see for example** [**https://osf.io/m4brf**](https://osf.io/m4brf)**. We agree that it would be interesting to perform regression analyses for further practices beyond timely publication of results. However, we consider these analyses beyond the scope of this manuscript which has the primary objective of presenting a dashboard to communicate transparency assessments to UMCs. We would also like to point out that while we did not examine the heterogeneity in the outcomes in study characteristics, the dashboard allows the data to be explored in different ways (e.g., filter by registry). The access credentials for the dashboard are now available directly in the manuscript.**

*4.	The authors have used a dataset, published previously, that includes trials from 2009-2017. The standards and cultural expectations of transparency, including registration and results reporting, ORCID ID associations, publication preprint and access, have changed significantly in the last 5 years. It is unclear, if this approach is to be scalable and replicable as claimed, why the data was not updated to current state or at a minimum, a more recent end date (July 2021). Only the metric for publications would need to be adjusted for time, given the 2 year window for publication. All other metrics could be shown. A 5 year delay (or, as apparent annual updates are planned that include data from 3 years prior) is hardly relevant to current activities either by institution or sponsor.*

**Author response: We thank the reviewer for this comment. Trials in the cohort used in this study had 2017 as the most recent completion year to allow for at least 3-5 years follow-up for the publication search. Given the relatively low rates of timely reporting (<2 years after trial completion), these longer follow-up periods are informative for whether any results were reported beyond that time period. The publication search requires resource-intensive manual checks, therefore most publication-based metrics are more challenging to update on a continuous basis. However, we very much agree that the dashboard should continue to be updated; we are planning an update of the cohort with trials completed 2018 - 2021. Moreover, in an effort to reflect the most up-to-date status for registration-based metrics (prospective registration, summary results reporting, publication link in registry), we updated the dashboard with the most up-to-date registry data we could obtain (query date: 1 November 2022). We also re-queried the Unpaywall API to get an up-to-date OA status of publications, which is also subject to change (query date: 1 November 2022). We have added more information on this in the Discussion: “We are planning regular updates of the registry data for trials already in the dashboard, as well as the inclusion of more recent cohorts of trials with at least 2 years follow-up (e.g., trials completed 2018-2021 assessed in 2023).” and “While the generation of the underlying dataset of clinical trials and associated results publications involves manual checks (approximately 10 person-hours per 100 trials), the assessment of transparency practices is largely automated.”**

a. *It is unclear why results reporting at 5 years is less than at 2 years. If a trial is reported within 2 years, isn't it also included in the 5 year number? It is certainly reported at 5 years if reported at 2. I suggest the authors update the data files and statistics, and replace the results as discussed below.*

**Author response: We thank the reviewer for pointing this out. Of course the 5-year publication rate is higher. This is an unfortunate consequence of the live dashboard not being accessible during review. We added toggles to the plots in the dashboard to visualize the data in different ways, including the results reporting plots. The screenshot in Fig 2 (now Fig 3) showed the 2-year reporting rate for reporting as a ‘manuscript publication only’, whereas it showed the 5-year reporting rate for ‘summary results only’. Thus, the lower reporting rate at 5 years simply reflected the visualization of different reporting routes (and rates for summary results reporting are generally low). We have replaced Fig 2 (now Fig 3) with an updated screenshot of the version in which the toggles are set to the same reporting route (‘summary results or manuscript publication’).**

5. *Similarly, for prospective registration of clinical trials, the authors used "a more recent cohort of interventional trials" 2006-2018. The field has changed dramatically since then, so why not use a truly recent cohort? E.g. ending 2021? Pooled statistics over that much time as described in the manuscript is not helpful.*

**Author response: The publication search was performed on the IntoValue cohort of trials completed 2009 - 2017. However, at the time of developing this dashboard we also had at our disposal a more recent cohort of interventional clinical trials registered in ClinicalTrials.gov with a start date between 2006 and 2018 and led by a German UMC. As prospective registration does not rely on the publication search, we decided to use this more recent cohort for the assessment of prospective registration in ClinicalTrials.gov (only). For parity with the IntoValue cohort, we filtered this more recent dataset for trials that were considered as complete based on study status in the registry. The development of an even more recent cohort would require a manual check of all trial affiliations to confirm that the trials were conducted by a German UMC. This manual check is resource-intensive and was not feasible for this revision. As mentioned in the previous comment, we agree with the reviewer on the importance of the dashboard being up-to-date. While it was not feasible to develop more recent cohorts for this revision, in an effort to address the reviewer’s comment we updated the dashboard with the most up-to-date registry and open access data we could obtain (see also comment above).**

6. *A major deficiency of the manuscript is the failure to describe the trends in the reported outcomes (trial registration, results reporting, linkage of registration to publications, etc.) as the substance of the results section. There have been significant changes since 2006; pooling all the results necessarily decreases the utility of the findings. Coupled with the lack of recent data, this is highly problematic. The data should be reported by year, and by institution, and interpreted at more granular level of analysis. Graphs are shown in the figures, but the trends are not the focus of the results described.*

**Author response: We very much welcome the reviewer’s comment. We hope that this is largely addressed by having access to the dashboard in the next round of revision. One of the core strengths of the dashboard is the visualization of different trends and the possibility of exploring the data (see drop-down menus in the dashboard). A paper is less well suited to describe the substantial number of trends across practices and institutions. To address this comment, we have also revised the Results section to emphasize the key trends for each practice.**

7. *The authors describe variability between and among UMC for trial registration but do not investigate that further. Are the trials that are not 'pre' registered applicable clinical trials? Are the trials not registered on Clinicaltrials.gov nevertheless registered on DRKS? What are the implications of interventional trials that are registered, but not pre-registered—did they need to be preregistered? Is the coding correct?*

**Author response: We thank the reviewer for raising this important point which we would like to clarify. In this and related studies, we used the WHO definition of a clinical trial, i.e., “any research study that prospectively assigns human participants or groups of humans to one or more health-related interventions to evaluate the effects on health outcomes” (**[**https://www.who.int/clinical-trials-registry-platform/about/glossary**](https://www.who.int/clinical-trials-registry-platform/about/glossary)**). Trials displayed in the dashboard include all studies that also meet our inclusion criteria (interventional, registered in ClinicalTrials.gov or DRKS, led by a German UMC, considered as complete between 2009 and 2017 per the registry). Thus, this dataset is not limited to (but may include) Clinical Trials of an Investigational Medicinal Product (CTIMPs) regulated by laws. We are only assessing compliance with legal requirements for summary results reporting in EudraCT, which we obtain from the EU Trials Tracker (see first plot under “Trial Reporting” in the start page of the dashboard). Where a trial is not regulated by law, trial sponsors still have a moral obligation to register clinical trials prospectively (Declaration of Helsinki) and the trial’s results should be reported in line with established guidelines (e.g., WHO, ICMJE, CONSORT). The dashboard evaluated adherence to these guidelines. We have revised the Methods to highlight this point: “In this study, we focused on adherence to ethical principles and reporting guidelines that apply to all trials. Compliance with a legal regulation was only assessed for summary results reporting in the EUCTR.”**

8. *The authors claim that this manuscript describes "stakeholder engagement to provide UMCs with actionable information on trial transparency in an accessible and interactive format, and thereby empower their efforts to support improvement." This is not described in the current manuscript. Stakeholder engagement and how the stakeholders used or did not use the data to understand or change current practices are not described.*

**Author response: We thank the reviewer for highlighting this potential misunderstanding. We were referring to a former interview study conducted in our research group that informed the development of the dashboard. Stakeholders (UMC leadership, support staff, funders, and experts in responsible research) were invited to give their views on a prototype dashboard at the level of UMCs with metrics for responsible research (**[**https://journals.plos.org/plosone/article?id=10.1371/journal.pone.0269492**](https://journals.plos.org/plosone/article?id=10.1371/journal.pone.0269492)**). For example, stakeholders examining the prototype dashboard expressed difficulties in understanding the methods by which the metrics were compiled and the limitations inherent to these methods. This led to the inclusion of a widget for each plot describing the relevant limitations to consider, as well as mouse-overs showing the numerator and denominators in each plot. We recognize that the way in which this sentence was phrased could be misinterpreted and have now removed this sentence. We further clarified the stakeholder interview study in the Methods: “The dashboard was shaped by interviews with UMC leadership, support staff, funders, and experts in responsible research who provided feedback on a prototype version [40]. This feedback led to the inclusion of several features to facilitate the interpretation of the data and contextualize the practices included.”**

9. *The authors should appreciate that some of their suggestions are not accomplished with "minimal effort" as claimed. A simple update (retrospectively linking publications in the trial registration) in Clinicaltrials.gov "opens" the trial record, and the entire record must then be updated to current requirements. The number of changes to requirements and the effort to do so are significant and a disincentive to retrospective changes.*

**Author response: We thank the reviewer for this very important point. We removed the “minimal effort” language and revised the relevant section in the manuscript accordingly: “Some of the practices included in this dashboard can still be addressed retrospectively, such as linking publications in the trial registration (realized for 49% of trials with a publication).”**

10. *Note that* [*https://quest-cttd.bihealth.org*](https://quest-cttd.bihealth.org) *requires a log in, which, given confidentiality provisions for reviewers, is not possible. The manuscript does alert to the fact that it will be open access upon publication, but it makes it difficult to review the utility, user-experience, and impact in the absence of access. Authors' claims to its novelty and importance (e.g., "provid[ing[actionable information") cannot be evaluated.*

**Author response: We are sorry to hear that the dashboard was not accessible. We now provide the credentials to access in the dashboard directly in the manuscript. The dashboard will be fully publicly available upon publication in a journal.**

## Reviewer #4:

*This is a useful and interesting article on a novel Dashboard that provides seven parameters on the quality of trials registration and reporting for German University Medical Centres (UMCs). The centrepiece of the article is really the Figure to showing the Dashboard for all UMC's. However, the text also provides some interesting and novel data on registration and reporting features, such as the proportion of published trials that report the trial registration number in both abstract and text, and the number of published trials which have provided a summary on the trials registry - which is appallingly low. While the article made interesting reading, I was left wondering what those in the University medical centres might think of this dashboard? Some suggestions for improvement:*

1. *It would help readers if the numbers in the text were more clearly linked to the Dashboard in Figure 2. For example,*

** line 178 Describes the 74% of trials are prospectively registered and*

** line 185 the 38% that have the trial registration number in the abstract.*

*These are the first two tiles of the Figure 2 Dashboard, and could be explicitly referred to in the text to help the reader link the text and the dashboard.*

**Author response: We thank the reviewer for this comment. We included Figure 2 to provide a broad overview of how the dashboard is structured, and all the data can be visualized in full directly in the dashboard. We were sorry to hear that the dashboard was not accessible during the review. We now provide the credentials to access in the dashboard directly in the manuscript. The dashboard will be fully publicly available upon publication in a journal. To address this comment, we now explicitly refer to Figure 3 (previously Figure 2) in the Results section.**

2. *I was puzzled that 44% of trials had published within two years but only 7% within five years - which seems contradictory. Can the authors explain this please?*

**Author response: We thank the reviewer for pointing this out. Of course the 5-year publication rate is higher. This is an unfortunate consequence of the live dashboard not being accessible during review. We added toggles to the plots in the dashboard to visualize the data in different ways, including the results reporting plots. The screenshot in Fig 2 (now Fig 3) showed the 2-year reporting rate for reporting as a ‘manuscript publication only’, whereas it showed the 5-year reporting rate for ‘summary results only’. Thus, the lower reporting rate at 5 years simply reflected the visualization of different reporting routes (and rates for summary results reporting are generally low). We have replaced Fig 2 (now Fig 3) with an updated screenshot of the version in which the toggles are set to the same reporting route (‘summary results or manuscript publication’).**

3. *I was expecting and hoping that the Discussion would explore some of the practicalities and barriers for usage in practice by the UMCS. But there seemed to be little on this. Some of the questions I would like to see answered are:*

*Q1. How much manual work was required to produce the dashboards (that is the number of person-months or costs for doing this) and how much might that be for an annual process?*

**Author response: The main steps of our approach that require manual work include the publication search and the validation of affiliations associated with clinical trials. The resources for developing a dataset for clinical trials as reflected in this publication depends on the number of included trials per UMC. For 100 trials, we estimate approximately 10 hours of work for someone who knows how to use ClinicalTrials.gov, Pubmed, and Google Scholar. Our methods have been successfully applied in other contexts (e.g., Poland,** [**https://bmjopen.bmj.com/content/10/1/e034666**](https://bmjopen.bmj.com/content/10/1/e034666)**), and we are currently collaborating with other international groups (USA, Sweden) that aim to develop similar trial transparency datasets and display them via an adapted version of our dashboard (e.g., protocol:** [**https://osf.io/u9d5c/**](https://osf.io/u9d5c/)**). To address the reviewer’s comment, we added a Supplemental file that provides an overview of the steps needed to produce such a dashboard, and their dependency on automated vs. manual methods (see Supplement S1). We also refer to this Supplement in the Methods: “An overview of the dependence of these steps on automated vs manual approaches is provided in Supplement S1.” and in the Discussion: “The datasets and methods used in this study can be scaled: this has been demonstrated in another European country (Poland) [49] and is currently underway in California, USA [50]. While the generation of the underlying dataset of clinical trials and associated results publications involves manual checks (approximately 10 person-hours per 100 trials), the assessment of transparency practices is largely automated. Institutions in possession of an in-house cohort of clinical trials registry numbers and persistent identifiers (e.g., DOIs) from matched journal publications, however, could achieve results more quickly. The code to create the dashboard is openly available and can be adapted to other cohorts.”**

*Q2. Has the dashboard been actively used by any current UMC's (eg the author's institution?) and what has been their reaction to this?*

**Author response: We thank the reviewer for this question. While the dashboard will be available to German UMCs only after the publication of this paper, they have been consulted in the process of developing the dashboard and underlying dataset. We have already presented the dashboard in the form of preliminary results to several research deans from German UMCs in the context of the association of medical faculties in Germany (Medizinischer Fakultätentag, MFT). The development of the dashboard was also informed by interviews with representatives of German UMCs (e.g., UMC leadership, support staff) to get their views on a prototype dashboard with metrics for responsible research (**[**https://journals.plos.org/plosone/article?id=10.1371/journal.pone.0269492**](https://journals.plos.org/plosone/article?id=10.1371/journal.pone.0269492)**). The dashboard will be made live and actively available to all German UMCs alongside the greater public upon publication. We then plan to organize a workshop with interested UMC parties once the dashboard is live. We believe that a peer-reviewed publication is an important element to improve the uptake of the dashboard among German UMCs.**

*Q3. Clinical trials are important but small proportion of all clinical research, and that proportion will vary widely across UMCs. Might this be included in the Dashboard all the reports to UMC's, and/or are their plans for extending the range of reporting to other types of studies?*

*Such questions might be usefully addressed in the Discussion.*

**Author response: We welcome this comment. We are eager to expand these efforts to other types of research (e.g. observational clinical studies) as well as other aspects beyond transparency (e.g. robustness or relevance indicators). In the Discussion we mention further development of the dashboard approach towards the broader category of clinical research and the inclusion of further metrics. It now reads: “We are planning regular updates of the registry data for trials already in the dashboard, as well as the inclusion of more recent cohorts of trials with at least 2 years follow-up (e.g., trials completed 2018-2021 assessed in 2023). Beside updates further transparency metrics may be integrated into the dashboard in the future, e.g., dissemination of results as preprints, adherence to reporting guidelines [3], or data sharing [52]. Beyond transparency, other potential metrics for a dashboard could reflect the number of discontinued trials [53] or the proportion of trials that inform clinical practice [54]. The development of such metrics should acknowledge the availability of standards and infrastructure pertaining to the underlying practices [23] and differences between study types and disciplines [27]. Future versions of the dashboard may also display additional sub-population comparisons, such as different clinical trial registries or UMC particularities [55].”**
