# Supplementary material for: Institutional dashboards on clinical trial transparency for University Medical Centers: A case study
Source: PLoS Med. 2023 Mar 21;20(3):e1004175. doi: 10.1371/journal.pmed.1004175 (PMC10030018; doi:10.1371/journal.pmed.1004175)
Supplement: Attachment — Submitted filename: response-to-reviewers.docx [file pmed.1004175.s012.docx]

# Response to editor and reviewers

**Manuscript number**: PMEDICINE-D-22-01430R2

We are grateful to the editor and reviewers for their remaining comments.

Below we provide a point-by-point response to each of the comments and highlight the corresponding changes we have made to the manuscript in **blue** text.

We would also like to highlight the following changes to the references:

- We added a reference for further transparency metrics that may be integrated in the dashboard in the future (see lines 418-419 in clean version).
- We found one instance of a citation to both the preprint and published version of the same paper. We removed the citation to the preprint and preserved the citation to the full paper (reference 10).

## From the Academic Editor:

1. *Please revise your title to “Institutional dashboards on clinical trial transparency for University Medical Centers: A case study”.*

**Author response: We thank the editor for this suggestion. We have changed the title accordingly: “Institutional dashboards on clinical trial transparency for University Medical Centers: A case study“.**

2. *Data Availability Statement: Please confirm the login requirements for the dashboard will be removed prior to publication.*

**Author response: We confirm that the login requirements for the dashboard will be removed prior to publication. In line with this, we have also removed the dashboard credentials from the main text of the manuscript.**

3. *Please trim your Author Summary such that it contains 2-3 single sentence bullet points under each of the three questions.*

**Author response: Following the editor’s suggestion, we have trimmed the Author Summary to 2-3 single sentence bullet points under each of the three questions. The Author Summary now reads:**

**“Why was this study done?**

- **Clinical trials are the foundation of evidence-based medicine and should follow established guidelines for transparency: their results should be available, findable, and accessible regardless of the outcome.**
- **Previous studies have shown that many clinical trials fall short of transparency guidelines, which distorts the medical evidence base, creates research waste, and undermines medical decision making.**
- **University Medical Centers play an important role in increasing clinical trial transparency but are often unaware of their performance of these practices, making it difficult to drive improvement.**

**What did the researchers do and find?**

- **We developed a pipeline to evaluate clinical trials across several established practices for clinical trial transparency and applied it in a cohort of 2,895 clinical trials led by German University Medical Centers.**
- **We found that while some practices are gaining adherence (e.g., prospective registration in ClinicalTrials.gov increased from 33% to 75% over the period considered), there is much room for improvement (e.g., 41% of trials reported results within 2 years of trial completion).**
- **We developed a dashboard to communicate these transparency assessments to University Medical Centers and support their efforts to improve.**

**What do these findings mean?**

- **Our study demonstrates the feasibility of developing a dashboard to communicate adherence to established practices for clinical trial transparency.**
- **By highlighting areas for improvement, the dashboard provides actionable information to University Medical Centers to empower their efforts to improve.**
- **The dashboard may inform interventions to increase clinical trial transparency and be scaled to other countries and stakeholders, such as funders or clinical trial registries.”**

4. *Please include key numbers in your Author Summary such as sample size and headline results.*

**Author response: We thank the editor for this comment. We included the sample size and two key numbers as headline results in the Author Summary. Given the focus of this paper on the feasibility of generating such a dashboard, the breadth of results displayed therein, and the space limits of the Author Summary, it was not possible to appropriately include further headline results relating to the uptake of specific practices.**

5. *Figure 3, while suitable for showing the dashboard home page, is not ideal for graphically representing the key findings (the individual panels are too small to appreciate, and axis/data labels are difficult to read). I would suggest providing screenshots of the individual panels in the Supporting Information and citing these in the Results instead. Figure 3 can still be kept in the main body of the manuscript but only cited when referring to the dashboard home page at line 347.*

**Author response: We agree with the editor that while Figure 3 is appropriate to provide a general overview of the dashboard Start page, screenshots of individual panels (or indeed the dashboard itself) are better suited to examine the results in more detail. We have kept Figure 3 in the main body and have moved it to after the only time it is cited, under “Interactive dashboard” (line 339 in clean version). We also created an additional Supplement, Supplement S9, which contains screenshots of the individual panels referred to in the Results (the previous Supplement S9 is now Supplement S10).**

# Comments from the reviewers:

## Reviewer #1:

1. *I thank authors for their diligent work in revising the manuscript and addressing the reviewers' questions. I do not have any additional comments.*

**Author response: Thank you!**

## Reviewer #2:

1. *The authors have responded to all my comments adequately. One minor comment whiie their response to comment 2 about  CT.Gov was fine I wanted to make sure they take care to consider carefully the denominators when comparing platforms.*

**Author response: We thank the reviewer and acknowledge this important point.**

## Reviewer #3:

1. *The authors have spent considerable time and effort responding to the suggestions of the editors and the reviewers. This reviewer believes that the authors should be credited with their attention to detail, their own transparency in the process, and the general management of the submission.*

**Author response: Thank you!**
